# Exploiting Molecular Dynamics in Composite Coatings to Design Robust Super‐Repellent Surfaces

**DOI:** 10.1002/advs.202104331

**Published:** 2022-01-07

**Authors:** Rui Guo, Eirini Goudeli, Wanjun Xu, Joseph J. Richardson, Weijian Xu, Shuaijun Pan

**Affiliations:** ^1^ State Key Laboratory of Chemo/Biosensing and Chemometrics and College of Chemistry and Chemical Engineering Hunan University Changsha 410082 China; ^2^ Department of Chemical Engineering The University of Melbourne Parkville Victoria 3010 Australia; ^3^ Department of Materials Engineering The University of Tokyo 7‐3‐1 Hongo, Bunkyo Tokyo 113‐8656 Japan

**Keywords:** droplet bouncing, molecular dynamics simulations, polymeric binding, repellent coatings, surface engineering

## Abstract

Fluorinated motifs are promising for the engineering of repellent coatings, however, a fundamental understanding of how to effectively bind these motifs to various substrates is required to improve their stability in different use scenarios. Herein, the binding of fluorinated polyhedral oligomeric silsesquioxanes (POSS) using a cyanoacrylate glue (binder) is computationally and experimentally evaluated. The composite POSS–binder coatings display ultralow surface energy (≈10 mJ m^−2^), while still having large surface adhesions to substrates (300–400 nN), highlighting that super‐repellent coatings (contact angles >150°) can be readily generated with this composite approach. Importantly, the coatings show super‐repellency to both corrosive liquids (e.g., 98 wt% H_2_SO_4_) and ultralow surface tension liquids (e.g., alcohols), with ultralow roll‐off angles (<5°), and tunable resistance to liquid penetration. Additionally, these coatings demonstrate the potential in effective cargo loading and robust self‐cleaning properties, where experimental datasets are correlated with both relevant theoretical predictions and systematic all‐atom molecular dynamics simulations of the repellent coatings. This work not only holds promise for chemical shielding, heat transfer, and liquid manipulations but offers a facile yet robust pathway for engineering advanced coatings by effectively combining components for their mutually desired properties.

## Graphical abstract

Super‐repellent nanocomposite coatings, consisting of low‐energy fluorinated motifs and adherent polymeric binders, can undergo rapid assembly and anchor to various substrate, displaying robust and durable resistance to a wide spectrum of liquids (including corrosive liquids and low surface tension liquids), with the super‐repellency strongly correlating to the physiochemical properties of the interface demonstrated both experimentally and theoretically.

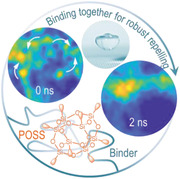

## 1 Introduction

The interfacial design of materials to achieve desirable macroscopic properties is of significance in the engineering of advanced functional surfaces. Surfaces and interfaces that maintain high‐performance or sustained stability after contacting small droplets, bulk liquids, or impinging fluids have found extensive potential in diverse applications including power generation,^[^ ^1^ ^]^ high‐speed wrapping and assembly,^[^ ^2^ ^]^ solvation or dehydration,^[^ ^3^ ^]^ membrane separations and conductions,^[^ ^4^ ^]^ biomechanics and robotics,^[^ ^5^ ^]^ self‐cleaning,^[^ ^6^ ^]^ and antifouling and accretion control.^[^ ^6^, ^7^ ^]^ Specifically, repellent surfaces are a class of surfaces featuring nonwetting/nonfouling properties, which can find extensive applications in fields requiring reduced drag, low‐fouling, enhanced cargo loading, and/or controlled liquid transportation, e.g., the exploration of oceans and their resources using realiable carriers with high‐performance repellent coatings.^[^ ^6^, ^7^ ^]^ Recent advances in designing super‐repellent surfaces (i.e., high‐performance repellence categorized by ultrahigh contact angles (>150°) and ultralow roll‐off angles (<10°)) include modifications to surface chemistry^[^ ^8^ ^]^ and/or surface structures,^[^ ^9^ ^]^ which can be achieved through bottom‐up (e.g., coating) or top‐down (e.g., etching) pathways. Bottom‐up approaches like spraying^[^ ^6^, ^10^ ^]^ are more substrate‐independent, especially when depositing coatings that utilize rapid interfacial polymerization. Generally, the integration of low‐energy motifs, e.g., fluorinated molecules^[^ ^6^, ^8^, ^11^ ^]^ and fluorinated nanoparticles,^[^ ^8^, ^12^ ^]^ allows for the incorporation of super‐repellency to a wide spectrum of liquids to the coatings. However, binding fluorinated materials like Teflon to substrates is particularly challenging due to the low‐surface energy and incompatibility between the coating components (e.g., phase separation and/or degradation/detachment of active motifs upon repetitive usage),^[^ ^3^, ^10^, ^13^ ^]^ and therefore, an in‐depth understanding at the molecular level of composite binders and low‐energy component systems is essential for a better design of such repellent coatings. Moreover, the stability of repellent coatings—an important factor in practical applications^[^ ^14^ ^]^—requires a deep understanding at the macroscopic (structural damage upon external stimuli^[^ ^15^ ^]^), microscopic (microstructural durability), and molecular (chemical stability) levels with the latter two points (i.e., interfacial molecular dynamics) largely unexplored in the literature.^[^ ^3a^ ^]^ We envision that exploiting molecular dynamics in composite coatings to create durable yet super‐repellent coatings and investigating their chemical mechanics, interfacial dynamics, and correlations with practical performance are fundamental in the surface engineering of advanced coating materials for various applications.

A particularly promising precursor for studying high‐performance coatings is polyhedral oligomeric silsesquioxanes (POSS)—a cubic rigid structure with the central inorganic cage grafted with organic motifs at the eight vertices.^[^ ^16^ ^]^ POSS‐based materials, as versatile building blocks, can be used in creating stable thin films on (either organic or inorganic) planar substrates and nanoparticles,^[^ ^16c^ ^]^ and are also useful for creating nanoporous surfaces and interfaces,^[^ ^16d^ ^]^ holding promise in diverse applications.^[^ ^16e^ ^]^ Specifically, fluorinated POSS (e.g., fluorodecyl POSS, substituted by fluorinated alkanes) is a class of low‐energy materials^[^ ^8^, ^10^, ^17^ ^]^ repellent to most liquids that still display excellent chemical and thermal stability, and potentially could be integrated with polymeric binders^[^ ^16f–h^ ^]^ to generate super‐repellent coatings.

**Figure 1:**
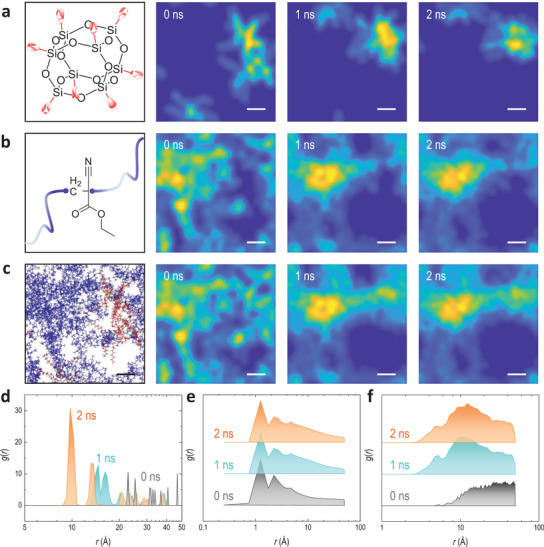
Molecular dynamics of the POSS–binder system. a–c) Chemical structures of POSS a), binder (i.e., poly(ethyl cyanoacrylate), pECA) b), and the initial configuration of a POSS–binder system containing 5 × POSS and 10 × pECA (with 50 repeating units) building blocks c). The red symbols in a) represent 1H,1H,2H,2H‐heptadecafluorodecyl substitutes (i.e., (CH_2_)_2_(CF_2_)_7_CF_3_). The blue lines in b) represent the chains of the polymeric binder. Their mass distributions (i.e., POSS, binder, and their composite) in the simulation cell (70 × 70 × 70 Å^3^) are shown as heatmaps (i.e., projected mass distribution) at different times in a–c), respectively. Blue‐to‐yellow (i.e., vacuum‐to‐mass aggregates) color coding is used. The mass aggregates (represented by yellowish domains) include both components (POSS and binder). The left panel of c) is the initial configuration, where the molecules of POSS and binder are presented by red and blue, respectively. Scale bars are 10 Å. d–f) Radial distribution functions (RDFs, *g*(*r*)) of POSS–POSS d), binder–binder e), and POSS–binder f) at different simulation times. These RDFs (apart from 0 ns) are time‐averaged over a period of 0.5 ns to show the variations in the structure, i.e., 1 ns corresponds to the time‐average RDFs from 0.75 to 1.25 ns, 2 ns corresponds to the time‐average RDFs from 1.75 to 2.25 ns.

**Figure 2:**
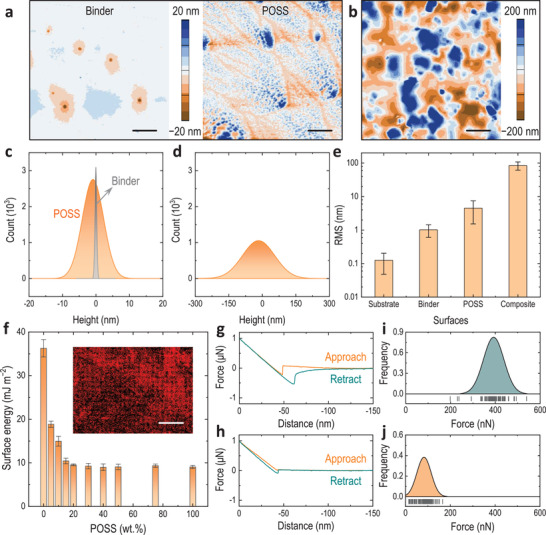
Flat composite coatings (20 wt% POSS) and their interfacial properties. a) AFM images of the binder (left) and the POSS (right) coated mica surfaces. Scale bars are 5 µm. b) AFM image of the composite (20 wt% POSS) coating on mica. The mica substrates used are 12 mm in diameter. Scale bar is 5 µm. c) Height histogram of the AFM images in a). d) Height histogram of the AFM image in b). The sample sizes are >60 000 and fitted using normal Gaussian distribution function in c) and d). e) Average RMS roughness of surfaces in a,b) and a mica substrate. Note that error bars of RMS roughness in this work are standard deviations of >5 measurements on different areas (2 × 2 µm^2^). f) Surface energies of POSS–binder coatings. Error bars are standard deviations of >3 measurements. The inset is an energy‐dispersive X‐ray spectroscopy (EDX) fluorine map of the coatings. Scale bar is 500 µm. g–j) Representative atomic adhesion force curves of the binder g) and annealed (200 °C) POSS surfaces (crystal region, i.e., smoother) h) determined by silica colloidal‐probe AFM, and their force (Gaussian) distributions of 100 measurements (at different spots) on the binder i) and the annealed POSS j) surfaces, respectively. The probes are 1 µm in diameter, with a nominal spring constant of 16 N m^−1^.

**Figure 3:**
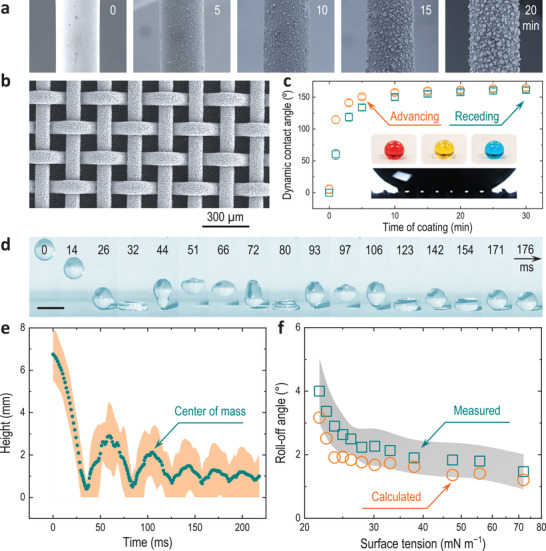
Spray coatings of super‐repellent POSS–binder. a) Scanning electron microscopy (SEM) images of spray coatings on stainless steel wires (diameter ≈500 µm) with different spray times (flow rate ≈1 mL min^−1^) using a solution containing 20 mg mL^−1^ pECA + 20 wt% POSS in 2,2‐dichloro‐1,1,1,3,3‐pentafluoropropane. b) SEM image of a typical super‐repellent copper mesh surface (≈10 min spraying each side). c) Dynamic contact angles of ethanol droplets (≈5 µL). Error bars are standard deviations of >3 measurements. The insets are liquid droplets beading up on the sprayed coating (top panel from left to right: *n*‐decane (red), ethanol (yellow), and water (blue)) and a silhouette image (bottom panel) showing the local contact (i.e., air pockets) of an ethanol droplet with the super‐repellent coating. d) Snapshots of an ethanol droplet (diameter ≈2.4 mm) bouncing on a super‐repellent surface placed horizontally. Scale bar is 3 mm. e) Bouncing trajectory of the droplet shown in d) as a function of time analyzed by FASTCAM Viewer 4 (PFV4). The top and the bottom height profiles of the bouncing droplet are indicated by the shaded area. f) Roll‐off angles of ≈10 µL droplets (water–ethanol mixtures). The shaded area represents the standard deviation of >3 measurements.

**Figure 4:**
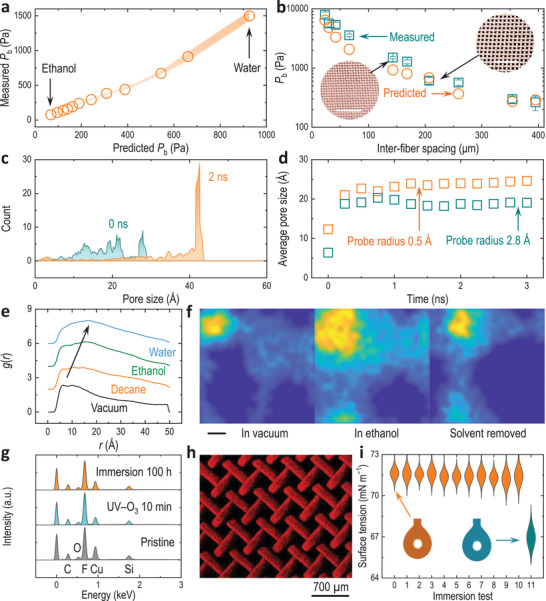
Stability of the super‐repellent coatings. a) Measured and predicted breakthrough pressures (*P*_b_) of water–ethanol mixtures on a coated super‐repellent surface (100 × 100 copper mesh, i.e., number of openings per 1 × 1 in^2^). Standard deviations of >3 measurements are presented by the shaded area. b) Water *P*_b_ on the coated mesh surfaces with different inter‐fiber spacings. The insets are photos of the coated 80 × 80 (left) and 60 × 60 (right) copper meshes (scale bars are 5 mm). The errors are standard deviations of >3 measurements. c,d) Pore size distributions assessed by MD simulations c) (probe radius 0.5 Å) of the model POSS–binder system and d) the pore size evolutions evaluated by different probe sizes. The sample size in c) is 2000. The pore sizes in d are the average of 2000 measurements performed at a radial distance step of 0.05 Å in the range of 0–70 Å. e,f) Stability in solvents evaluated by MD simulations: *g*(*r*) of fluorine–binder e) (200 solvent molecules) and the projected mass distributions f) (heatmaps; 6400 solvent atoms). The simulation system in c–f) consists of 5 × POSS + 5 × pECA (*n* = 50) with a solid density of 0.5 g cm^−3^. The dimension of the heatmaps is 70 Å × 70 Å. Blue‐to‐yellow (i.e., vacuum‐to‐mass aggregates) color coding is used in f). g) EDX spectra of super‐repellent coatings subjected to different treatments (i.e., 100 h immersion in ethanol, 10 min exposure to UV–O_3_). h) EDX mapping of fluorine moieties after immersion in ethanol for 100 h. i) Violin plot of water surface tensions after 10 min incubation with the super‐repellent coatings (subjected to different treatments). X‐axis represents the type of treatment, i.e., 0 = fresh Milli‐Q water control (no immersion/substrate involved); 1 = fresh super‐repellent coating without treatment; 2 = 5 min heat treatment at 150 °C; 3 = 20 min UV–O_3_ treatment; 4 = 60 s air plasma treatment; 5 = tape peeling (applied force ≈10 N) test; 6 = 10 min immersion in ethanol; 7 = 10 min immersion in *n*‐decane; 8 = 10 min immersion in dimethylformamide; 9 = 10 min immersion in 98 wt% H_2_SO_4_; 10 = 10 min immersion in ≈19 m NaOH; 11 = noncoated mesh control (i.e., hydrophilic) immersed in ethanol for 10 min. The insets are the pendent drops of water (≈7 µL) after 10 min contact with the surface treated correspondingly. Data box of at least 10 measurements (sample size) for each scenario are shown as normal symmetric distribution curve (the significant differences are not assessed).

**Figure 5:**
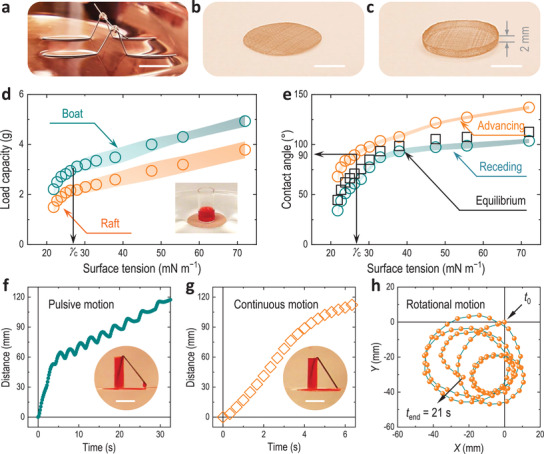
Floating, loading, and locomotion of super‐repellent surfaces on liquid. a–c) A super‐repellent stainless steel “strider” (wire diameter 1 mm) a) floating on an oil surface (*n*‐hexadecane), and super‐repellent copper mesh “raft” b) and “boat” c) with a height of ≈2 mm. Scale bars are 1 cm. d) Load capacity of the super‐repellent coatings as a function of the liquid underneath. The inset is to illustrate the measurement, and the total weight (i.e., the container, the added liquid, and the raft) when falling into the pool (water–ethanol mixtures) is recorded. e) Measured dynamic contact angles and calculated equilibrium contact angles of water‐ethanol mixtures (≈5 µL) on silicon wafers coated with pECA + 20 wt% POSS (20 mg mL^−1^ in 2,2‐dichloro‐1,1,1,3,3‐pentafluoropropane) by drop casting. Shaded areas in (d and e) represent standard deviations of >3 measurements. f–h) Pulsive (intermittent) f), continuous g), and h) rotational locomotion of a super‐repellent raft on the water surface powered by loaded liquid “fuel” (i.e., ethanol). The insets are the snapshots of the pulsive locomotion f) (needle suspended) and the continuous linear locomotion g) (needle immersed), where ethanol is colored red to differentiate from the liquid in the pool. Scale bars of the insets are 1 cm.

Herein, we use a two‐component coating system containing a polymerizable binder (i.e., ethyl cyanoacrylate, ECA) with a high binding affinity to different substrates through either polar or nonpolar interactions and a low‐energy precursor (i.e., fluorinated POSS) as a model super‐repellent system for studying the mechanics of repellent coating systems via both all‐atom molecular dynamics (MD) simulations and systematic experimental approaches at different scale levels (**Figure** **1**). The POSS precursor is selected not only because of its low‐energy^[^ ^17^ ^]^ but also because it serves as a nucleation agent for the formation of hierarchically rough porous structures^[^ ^10^ ^]^ that help maintain an air layer at the coating surface and thus contribute toward super‐repellency. The ECA monomer can undergo rapid polymerization (i.e., pECA) in the presence of moisture and provides adherence to nearly all substrates,^[^ ^18^ ^]^ thus serving as a promising binder to effectively and robustly anchor POSS in the coatings. The composite coatings drop‐cast on planar substrates (i.e., finer structure) display enhanced roughness, sustained low surface energy, as well as strong adhesion both to hydrophobic (e.g., polystyrene, PS) and hydrophilic (e.g., silica) substrates (**Figure** **2**). Moreover, upon high‐pressure spraying, the coating system nucleats and forms hierarchically re‐entrant microstructures on mesh substrates, displaying super‐repellency (e.g., ultrahigh contact angles, ultralow roll‐off angles, and droplet bouncing) even to ultralow surface tension liquids like alcohols—conventionally a complete wetting liquid on most surfaces (**Figure** **3**). The super‐repellent surfaces exhibit high breakthrough pressure (up to 10 kPa for water) and have tunable robustness via a careful choice of the mesh substrates. The stability of these coatings is further validated both by MD simulations (by adding solvent molecules) and different experimental approaches (e.g., immersion, peeling test, and thermal treatment) (**Figure** **4**). Interestingly, the super‐repellent mesh surfaces can float intact (e.g., without wetting or sinking) and can move with controlled linear or rotational locomotion continuously or in pulses on a series of liquid surfaces. The loading capacity of the locomotive super‐repellent surfaces is strongly correlated to the surface tension of the liquid (**Figure** **5**). The interfacial insights and structural correlations of the super‐repellent coatings studied in this work thereby provide fundamental implications for the future design of advanced surfaces and interfaces.

## 2 Results and Discussion

### 2.1 Molecular Dynamics Simulations

To assess the molecular and structural basis of the POSS–binder coatings, all‐atom MD simulations were carried out using a system consisting of 5 × POSS and 10 × pECA molecules (an arbitrary repeating unit *n* = 50 was used to represent the polymeric binder) in a simulation cell (70 × 70 × 70 Å^3^) at room temperature (298 K) in a vacuum (Figure 1; and Movie S1; “Molecular dynamics simulations” in Supporting Information). The MD simulation of the POSS–binder composite at comparable concentrations to our real‐world experiments (≈25 and 20 wt% POSS, respectively) served as a preliminary computational evaluation for the experimental design. The POSS (Figure 1a) and the binder (Figure 1b) rapidly associated with each other and reached equilibrium (<1 ns) due to their intermolecular and/or intramolecular potentials, forming phase separations and mass segregations (i.e., porous structures) in the simulation cell (Figure 1c). The interspecies radial distribution functions (RDFs, g(r)) for POSS–POSS (Figure 1d), binder–binder (Figure 1e), POSS–binder (Figure 1f), as well as the fluorine–binder and the fluorine–cage distances (i.e., between the fluorinated alkane chain and the central polyhedral cage consisting of Si—O bonds) (Figure S1, Supporting Information), were calculated. The POSS motifs, serving as low‐energy fillers in the system, evolved from an initial POSS–POSS interspacing of ≈23 Å (corresponding to the positions of the first‐nearest neighbors) at 0 ns to eventually a feature of ≈10 Å at equilibrium (i.e., 2 ns) (Figure 1d). The RDF of the binders (i.e., pECA) did not change as significantly as POSS, featuring a constant first‐nearest distance of ≈1 Å throughout the simulations suggesting it is a robust binder (Figure 1e). The RDF of POSS–binder was broad due to the physical crosslinking (i.e., through intermolecular interactions) of the polymeric binders, having a first‐nearest interspacing of ≈6 Å and a dominant interspacing around 10 Å soon after the commencement of the MD simulations (≥1 ns) (Figure 1f). In addition, the fluorinated chains of POSS were associated through dispersive intermolecular forces with the binders at a dominant interspacing of 10–20 Å (Figure S1a, Supporting Information). Due to the polymeric binding that confined the POSS during the process, the fluorinated chains of POSS displayed a relatively reduced freedom with slightly reduced distance (by ≈1 Å) between the centers of mass of the fluorine chains and the cages (Figure S1b, Supporting Information). Other systems with varied POSS‐to‐binder ratios also displayed similar results in terms of interspecies binding, mass distributions, and the formation of pore structures (Figures S2 and S3, Supporting Information). These findings from MD simulations indicate that, though the binder and the POSS have distinct natures, these two constituents can associate with each other and form a continuous but nanoporous framework driven by their dispersive interactions. It is noted that other POSS–binder systems (e.g., size, ratio) might be needed in future to draw more statistically relevant conclusions.

### 2.2 Physiochemical Properties of Flat Coatings

For a better understanding of the intrinsic interfacial properties of the POSS–binder composite, coatings with finer features were prepared by drop‐casting coating solutions on smooth mica surfaces followed by solvent evaporation and polymerization (Figure 2; “Finer coatings” in the Supporting Information). Atomic force microscopy (AFM) was used to characterize the height profiles of mono‐component coatings (binder and POSS; Figure 2a) and the composite coating (20 wt% POSS; Figure 2b). Distinct rough morphologies were observed on the composite coating (with a thickness of ≈400 nm; Figure S4, Supporting Information), compared to the mono‐component control surfaces. The normal Gaussian height histograms of their AFM images revealed that the height of the binder surface was distributed in a narrow region (±1 nm), followed by the POSS control (±10 nm) (Figure 2c), while the composite yielded a broadened distribution (±150 nm) (Figure 2d). Root‐mean‐square (RMS) roughness demonstrated that, upon coating with the POSS–binder composite, the smooth mica substrate (≈0.1 nm) was rendered significantly increased roughness (i.e., ≈1000 times rougher than the substrate) (Figure 2e). The enhanced roughness was assumed to be a result of phase separations as predicted by the abovementioned MD simulations, which provided a contributing structural factor for repellent surfaces (i.e., air trapping at rough surface^[^ ^19^ ^]^) in conjunction with the presence of the low‐energy POSS.

To determine an optimal composition for the repellent coatings, the surface energies of different POSS–binder composites were studied using the Owens–Wendt method^[^ ^20^ ^]^ (Figure 2f; “Surface energy” in the Supporting Information). The total surface energy of the composites decreased as the amount of low‐energy POSS was increased, and a minimum of ≈10 mJ m^−2^ was obtained when POSS was >10 wt%. That is to say, a small amount of low‐energy POSS can significantly reduce the overall surface energy of the composite, based on the minimum free surface energy principle, i.e., low‐energy motifs can migrate to the solid–air interface to minimize the overall energy of the coating^[^ ^3^, ^10^, ^21^ ^]^ (Figures S5 and S6, Supporting Information). It is noted that the ultralow surface energy of the POSS–binder composites (e.g., 20 wt% POSS) was dominantly dispersive (i.e., >98%) while the polar component only took up a negligible fraction (<2%) (Figure S7, Supporting Information).

To further investigate the interfacial forces of the composite coating, we studied their adhesion forces by colloidal‐probe AFM using either a hydrophobic or hydrophilic probe (1 µm in diameter). First, to minimize the roughness effect and to better reflect the intrinsic properties of the coatings (where roughness is minimized), the POSS surface was first annealed at 200 °C above its melting point^[^ ^17^ ^]^ (≈150 °C; Figure S8, Supporting Information). Upon annealing treatment, the RMS roughness of the POSS was greatly reduced, from ≈10 nm for a fresh surface (i.e., nonannealed) to ≈0.04 nm at crystalline regions or ≈0.36 nm at amorphous regions (Figure S9, Supporting Information) after annealing, which was comparable to that of an annealed binder surface (≈0.29 nm). POSS was stable during the annealing treatment as there was negligible weight loss (<0.2%; Figure S10, Supporting Information). Annealing treatment at 200 °C also did not change the surface of the binder (Figure S11, Supporting Information) or the composite surface (i.e., similar morphology and roughness) (Figure S12, Supporting Information), indicating their potential applications at high temperatures and that this polymeric confinement strategy could serve as a potential pathway for controlling the phase transitions of the POSS component. The adhesion forces of a hydrophilic silica probe (i.e., polar surface) with the binder and the annealed POSS crystal were 393 ± 46 and 83 ± 32 nN, respectively (Figure 2g–j), however, the amorphous region was relatively more adhesive (869 ± 300 nN) than the crystalline region, suggesting that the amorphous POSS coating was thin and the underneath mica surface (e.g., polar and hydrophilic) could interact with the probe (i.e., 2118 ± 69 nN) (Figure S13, Supporting Information). When using a less polar colloidal probe (e.g., polystyrene, PS), the probe–surface adhesion forces were 318 ± 44 and 210 ± 44 nN for the binder and POSS, respectively (Figure S14, Supporting Information). These results indicated that the polymeric binder was adhesive both to hydrophilic (e.g., silica) and hydrophobic (e.g., PS) surfaces, while the interaction forces of POSS with the probe surface were largely dispersive (i.e., a larger force with nonpolar PS vs smaller force with polar silica), further confirming its nonpolar, low‐energy nature. The adhesive property of the binder and the low‐energy property of the POSS together make the composite suitable for engineering substrate‐independent super‐repellent coatings.^[^ ^22^ ^]^

### 2.3 Spray Coatings and Super‐Repellency

We sprayed the POSS–binder composite (20 mg mL^−1^; 20 wt% POSS), using a spray gun powered by compressed air, onto mesh substrates that consisted of woven textures of metal wires (Figure 3; “Coarser coatings” in the Supporting Information). As shown in Figure 3a, hierarchically structured coatings were formed on the surface of the metal wires in 10–20 min, at a given spray rate of ≈1 mL min^−1^. The airflow‐driven spraying produced a uniform coating on the metal wires of a copper mesh substrate without blocking the inter‐fiber spacing, thereby allowing for the pores to reduce solid–liquid contact (Figure 3b). The effect of the coating time on the surface wettability was studied by dynamic (i.e., advancing and receding) contact angles using a low surface tension liquid that can wet most of the existing surfaces, namely ethanol. Both dynamic contact angles increased to >150° for coatings sprayed for ≥10 min with the contact angle hysteresis (CAH, difference between advancing and receding contact angles) generally less than 5°, indicating the coatings’ super‐repellency even to ultralow surface tension liquids (Figure 3c). The surface resisted successive impingements of an ethanol droplet (diameter ≈2.4 mm) released at a height of ≈7 mm above the surface (corresponding to an impacting velocity of 0.33 m s^−1^, or a total impact pressure^[^ ^23^ ^]^ of ≈80 kPa) without liquid penetration (or wetting) (Figure 3d; and Movie S2, Supporting Information). As described in the bouncing height profile, four full bounces were observed (Figure 3e) with the contact time during each bounce in the scale of 19–24 ms (consistent with ethanol's characteristic inertia–capillary timescale,^[^ ^2b^ ^],^ i.e., 22 ms for an ethanol droplet of 2.4 mm in diameter) (Figure S15, Supporting Information). This result indicated the adhesion force in the vertical direction between the probe liquid and the super‐repellent coating is negligible—consistent with the observation of the ultralow CAH (Figure S16, Supporting Information).

Roll‐off angles are another important parameter for characterizing repellent surfaces, with smaller values generally reflecting smaller lateral interaction (i.e., friction) between the droplet and the solid surface.^[^ ^24^ ^]^ By balancing the forces between the droplet gravity and the hysteresis acting at the advancing and receding air–solid–liquid triple‐phase contact line, roll‐off angles *ω* can be computed as^[^ ^25^ ^]^

(1) $$\omega \;=\arcsin\frac{2\gamma d\left(\cos\theta_{r}^{*}-\cos\;\theta_{a}^{*}\right)}{\pi \rho gV}$$

where *γ*, *ρ*, *V* are surface tension, density, and volume of the liquid droplet, respectively, $\theta_{a}^{*}$ and $\theta_{r}^{*}$ are advancing and receding contact angles of the spray coatings, *g* is the gravitational acceleration constant, and *d* is the diameter of the triple‐phase contact line dependent on the liquid volume and the apparent contact angle. The diameters of the region where the droplet sat on the coatings (i.e., *d*) for ≈5 µL water–ethanol mixtures (Figure S17, Supporting Information) were generally found in the range of 500–1000 µm (Figure S18, Supporting Information). As shown in Figure 3f, the measured roll‐off angles fit well with the calculated values and were all found to be <5° following the trend of liquid surface tensions. These ultralow CAH and roll‐off angles together demonstrate the ultralow solid–liquid interactions in both vertical and lateral directions and provide a basic understanding of the surface wettability where only surface tension and droplet gravity are involved (i.e., no external force applied).

### 2.4 Breakthrough Pressure

The stability and robustness of super‐repellent surfaces are an important factor leading to real‐world applications,^[^ ^6^, ^14^, ^15^ ^]^ and is therefore extensively investigated both at the macroscopic scale (e.g., breakthrough pressure, pealing test, thermal stability, immersion tests, and aging tests) and at the molecular scale by MD simulations (Figure 4; “Stability tests” in Supporting Information). First, the breakthrough pressure (*P*_b_, the minimum hydraulic pressure of the probe liquid at the coating surface that forces the liquid breaking into the coating and thereby resulting in wetting transition, i.e., from nonwetting to wetting) of our super‐repellent coatings can be assessed as following^[^ ^6^, ^10^ ^]^

(2) $$P_{b}=\frac{4\gamma R\left(1\;-\;\cos\theta_{e}\right)}{D\left(D\;+\;2R\sin\theta_{e}\right)}\;$$

where *R* and *D* are the fiber diameter and the inter‐fiber spacing of the super‐repellent mesh surface, respectively. *P*_b_ can be determined by the maximum height of a liquid column accumulated on the super‐repellent surface which is sandwiched between two vertically placed cylinders. The *P*_b_ of water–ethanol mixtures were measured and found to be largely dependent on the surface tension of the probe liquid as expected, i.e., *P*_b_ was largest for water (i.e., ≈1000 Pa) and lowest for pure ethanol (i.e., ≈90 Pa), following the general trend predicted by this method (Figure 4a; and Figure S19, Supporting Information). In addition, *P*_b_ was also tunable by the inter‐fiber spacing of the mesh substrates, i.e., smaller spacing resulted in larger *P*_b_ (Figure 4b; and Figure S20, Supporting Information). It is noted that *P*_b_ is a measure of the robustness of the nonwettting state, which is laregely determined by the macroscopic pores rather than the microscopic pores (see Equation (2)). These results, therefore, provide a structural understanding (for given *θ*_e_) for engineering robust super‐repellency via controlling the macroscopic pore size.

### 2.5 Microscopic Pores

Next, the microscopic pore structures of the POSS–binder systems, either of the space between the composite aggregates or the characteristic cage of the POSS motifs, were evaluated at the molecular level by MD simulations either in the vacuum or in the presence of solvent molecules (polar vs nonpolar, water vs oil) (Figure 4c–f). As for the POSS–binder system (≈40 wt% POSS), its pore size was initially (at 0 ns) broad at roughly 10–30 Å, which quickly transformed into a stable configuration with a dominant pore size slightly over 40 Å at 2 ns (Figure 4c; and Figure S21, Supporting Information). Slight variances were observed when using different probe radius (0.5 and 2.8 Å) for determining the average pore size of the composite system, with bigger probes ending with a slightly smaller average value (i.e., 25 and 20 Å for 0.5 and 2.8 Å probes, respectively, compared to the initial average pore size of ≈10 Å) (Figure 4d; and Figure S22, Supporting Information). Note for reference, a probe radius of 2.3 Å corresponds to the van der Waals radius of N_2_ and is equivalent to N_2_ adsorption measurements, while 2.8 Å is the default value in MD simulations and a smaller probe (i.e., 0.5 Å) is used to capture the cage porosity of POSS molecules. In addition, by maintaining the density of the simulation cell (0.5 g cm^−3^), the composite systems with more polymeric binders (i.e., less POSS filler) resulted in smaller pore sizes (Figures S21–S23, Supporting Information). The nanopores help maintain the air at the surface for achieving better repellence (i.e., higher contact angles) and generate higher capillary pressure (i.e., antiwetting or resisting local breakthrough), but also may cause delocalization of the POSS–binder network through solvation, thereby leading to performance deterioration.

Therefore, we revisited the MD of the equilibrium network and added the same number (200) of solvent molecules (*n*‐decane, ethanol, or water) into the system. As the solvent's polarity increased, the interspecies of fluorine–binder, binder–binder, and POSS–binder moved further apart, while POSS–POSS shifted to a slightly closer distance and no change was found for fluorine–POSS (Figure 4e; and Figure S24, Supporting Information). Similar results were obtained when using the same number of solvent atoms (i.e., 6400 atoms equal to 200 *n*‐decane, 711 ethanol, or 2133 water molecules, respectively) (Figure S25, Supporting Information). It is noted that although delocalization occurred, the overall network structures and interspecies distances did not display significant changes in the solvents (Figure 4f; and Figures S26–S28, Supporting Information) and were capable of restoring the initial equilibrium as simulated in the vacuum (Figures S29 and S30, Supporting Information). Specifically, the dominant distances of the POSS–binder changed from 10 ± 1 Å in vacuum to 11 ± 5, 12 ± 3, and 17 ± 6 Å when in *n*‐decane, ethanol, or water, respectively, which all returned to 10 ± 2 Å when solvents were removed (Movies S3–S5, Supporting Information). Note that due to the broad distributions, here we used the error ranges defined in an arbitrary way, e.g., ≈10% of the peak value. These results indicated the POSS–binder system was stable with solvent molecules on the molecular level from the aspect of MD simulations.

### 2.6 Interfacial Interactions

In addition, to provide further insights into the local wetting properties (i.e., at the interface close to the three‐phase contact line), we calculated the intermolecular energies between the coating componnets and the solvent molecules (i.e., decane, ethanol, and water) (Figure S31, Supporting Information). The interaction energies of POSS–binder and POSS–solvent are comparable especially in nonpolar decane (about −400 kcal mol^−1^, i.e., dispersive interactions), in contrast to the smaller values (between −100 and −300 kcal mol^−1^) when polar water is involved. The solvent–binder interactions are more negative (between −1100 and −2200 kcal mol^−1^) indicating that the polymer binder exerts a higher attractive force on the solvent than the POSS does, following the order ethanol > water > decane. In addition, we also calculated the contact area between POSS and the binder when solvents were present (Figure S32, Supporting Information). We found that the contact area between the POSS and the binder was larger when decane (nonpolar) was the solvent, while smaller contact area (i.e., bigger POSS–binder distance) was observed when water (polar) was the solvents, which was also consistent with the above interaction energy calculations. The results indicated that the polymeric binder (pECA) shared both polar and nonpolar interactions, allowing for effective binding of the repellent motifs (POSS) within the network in one way, and in another also exposing the risk that the network could be potentially attacted by the surrounding solvent molecules (i.e., coating stability).

### 2.7 Coating Stability

We experimentally evaluated the stability and/or durability of our super‐repellent coatings using various approaches (Figure 4g–i). The surface chemistry did not change after being treated by long‐term immersion in ethanol (≈100 h) or exposure to UV–O_3_ (10 min) (Figure 4g), where a good coverage of the repellent POSS motifs was retained on the surface (Figure 4h), still allowing water droplets to roll off the coatings at a small tilt angle (e.g., <3°) (Figure S33, Supporting Information). The super‐repellent coatings can also resist corrosive liquids, including concentrated acids (32 wt% HCl, 98 wt% H_2_SO_4_) and concentrated base (≈19 m NaOH) (Figure S34, Supporting Information). Specifically, the coverage of the repellent coating on the substrate remained intact even after 10 min immersion in 98 wt% H_2_SO_4_ (Figure S35, Supporting Information). In addition, the composite coating was stable at high temperatures up to 150 °C owing to the outstanding thermal stability of POSS (Figure S36, Supporting Information). The mechanical stability was also assessed by a peeling test where no obvious structural changes were observed and droplets of water–ethanol mixtures could roll off the coatings easily given small tilt angles (<5°) (Figure S37, Supporting Information). In addition, 10 different treatments were carried out (as shown in Figure 4i) to further evaluate the coating stability. After these treatments, there was no obvious change in water surface tension (Figure 4i) or contact angles (Figure S38, Supporting Information), indicating that no obvious chemical leaching occurred. Moreover, no obvious staining was found on the super‐repellent coating even after being incubated in a fluorescent solution for 10 min (Figure S39, Supporting Information), further confirming the robustness of the super‐repellent coatings.

### 2.8 Cargo Loading Application

We further investigated the super‐repellency of the coated mesh substrate when external forces were applied such as the weight of the substrate and/or the weight of cargo loaded onto the mesh when static or during locomotion (Figure 5; “Cargo carriers” in Supporting Information). Due to the ultrahigh super‐repellency, coated stainless steel wires can even float on the surface of *n*‐decane (i.e., a low surface tension oil) (Figure 5a). To better quantify the loading capacity, coated copper mesh substrates of specific size and shape were studied (Figure 5b,c). While floating on the surface of a mixture of water and ethanol, weighing liquid (water) was added dropwise until the super‐repellent raft (1 cm in diameter) or boat (1 cm in diameter with a 2 mm wall) sunk into the pool, and the total weight (including the substrate, container, and water added) was recorded as their loading capacity (Figure 5d). The load capacities of both super‐repellent carriers were linearly correlated to the surface tension for liquids *γ* > *γ*_c_, or in other words, for liquids above a critical surface tension of ≈27 mN m^−1^. The boat generally had a higher capacity than the raft for identical conditions owing to the extra buoyancy generated due to the 2 mm wall. Force balance analysis indicated that the loading capacity^[^ ^26^ ^]^ was dependent on the position of the triple–phase contact line when sinking occurred (Figure S40, Supporting Information), and the contact line moved as load increased until the saddle point was reached. For a surface having local contact angles *θ*_e_ ≥ 90°, the contact line finishes when the capacity is reached at the upper point (i.e., surface tension forces direct fully upward). For other cases (i.e., *θ*_e_ < 90°), the contact line finishes when a local contact angle *θ*_e_ is reached (i.e., surface tension forces act only partially upward). To confirm these assumptions, we measured the intrinsic advancing (*θ*_a_) and receding (*θ*_r_) contact angles of water–ethanol mixtures on the drop‐cast POSS–binder coatings on smooth surfaces to represent the local (finer) dynamic contact (at the point of maximum loading), and computed their equilibrium contact angles (Figure 5e)^[^ ^27^ ^]^

(3) $$\theta_{e}=\;\arccos\left(\frac{k_{a}\cos\theta_{a}\;+\;k_{r}\cos\theta_{r}}{k_{a}\;+\;k_{r}}\right)$$

where *k*_a_ and *k*_r_ are coefficients related to the advancing and receding contact angles (see Equations (S3) and (S4) in the Supporting Information).^[^ ^27^ ^]^ It is noted that the calculated *θ*_e_ is only to gauge a possible equilibrium and does not reflect the true value. Among the three parameters (i.e., *θ*_a_, *θ*_r_, *θ*_e_), *θ*_a_ was consistent with the observation of *γ*_c_ (i.e., *θ*_a_ ≈90°). The local contact angles analysis, therefore, explains when a maximum loading can be achieved. It is noted that although the surface microstructure (e.g., fiber diameter, inter‐fiber spacing) has a great influence on the surface super‐repellency (e.g., hierarchical structures allow for more solid–air interface and thereby enhanced repellence^[^ ^6b^ ^]^), the surface loading capacity is less dependent on these parameters (Figure S41, Supporting Information) but is strongly correlated to the local wettability (Figure 5e).^[^ ^26^ ^]^ Nevertheless, the inter‐fiber spacing is usually determining the breakthrough pressure (i.e., the robustness) of the super‐repellent surfaces (Figure 4b).

In addition, three different types of locomotion (linear pulsive, continuous, or rotational motion) were demonstrated using these super‐repellent striders (Figure 5f–h; and Movies S6–S8, Supporting Information). A low surface tension liquid (ethanol) was loaded onto a super‐repellent strider floating on the water surface, where the ethanol can be released due to a siphon effect at the rear of the super‐repellent strider through a V‐shape capillary (Figure S42; “Locomotion” in the Supporting Information). Dependent on the end position of the capillary, the “fuel” liquid can be released intermittently or continuously, thereby both pulsive (i.e., capillary suspended) and continuous (capillary immersed) motions can be achieved solely driven by surface tension differences across the front and the rear of the carrier,^[^ ^28^ ^]^ while the direction of motion was arbitrary (or rotational) unless the carrier was confined in a narrow waterway. In addition, the motion parameters (e.g., velocity, the interval of propulsion) can be controlled by the capillary inner diameter and/or the amount/type of fuel loaded (Figures S43 and S44, Supporting Information) or by a magnet if using a magnetic carrier (data not shown). These motion styles are demonstrated more from a view of further exploring the surface super‐repellency (e.g., self‐cleaning, robustness, cargo loading, and driving force of motions) rather than the view of practical senarios (and/or the comparison with classical boats). Of note, the locomotion was unaffected even when low surface tension fuel liquid contaminated the waterway, suggesting that the super‐repellent coating holds potential for the protection of marine vehicles missioning in harsh conditions such as oil‐spilled waterways, or for the exploration of hydrocarbon lakes on Titan. Moreover, the super‐repellency, cargo loading, and sliding motion on liquid surface make the coating promising for relevant drag reduction applications.^[^ ^7^, ^29^ ^]^ Interestingly, if sinking occurred during motion, the strider surface was not necessarily wet by the pool liquid as the sunk carrier was usually found dry and intact. That is, no breakthrough (or wetting by liquid penetration) occurred during any of the cargo loading experiments (Figure S45, Supporting Information), while sinking was likely due to the instability caused by the ultralow CAH (e.g., low friction). It is noted that although further experiments (e.g., viscosity effect; Figure S46, Supporting Information) and theoretical calculations are needed to prove the potential cargo loading applications, the super‐repellent coatings and their structure–property correlation revealed should provide fundamental design principles for advanced applications in relevant fields.

## 3 Conclusion

We developed a coating pathway for engineering repellent coatings and surfaces using a universal binder and a low‐energy precursor. Systematic experimental and MD simulation approaches revealed that this two‐component coating system combines the beneficial characteristics of both precursors and is a promising approach for achieving durable super‐repellency on different substrates (e.g., stainless steel, copper, silicon, and mica). When sprayed onto woven mesh substrates, the super‐repellent surfaces are capable of resisting a series of liquid droplets, preventing their penetrations either in static (e.g., water ‐ethanol droplets), dynamic (e.g., impinging ethanol droplets), or bulk (e.g., liquid pools for floating raft/boat) liquid systems, while also providing a means for enhanced cargo loading using this composite coating. Theoretical analysis and correlations (roll‐off angles and breakthrough pressures) also matched well with the experimental observations, confirming the proposed local contact regime between the solid surface and the surrounding liquid. Negligible changes of microstructures and POSS–binder associations were observed by simulations and also confirmed by a range of durability tests. These coatings and the results reported herein have extensive implications for the engineering of next‐generation advanced coatings and their on‐demand applications, e.g., wetting‐assisted patterning,^[^ ^30^ ^]^ supramolecular complexation,^[^ ^31^ ^]^ heterogeneous structuring,^[^ ^22^, ^32^ ^]^ droplet dynamics control,^[^ ^33^ ^]^ surface reinforcement and surface‐enhanced sensing,^[^ ^34^ ^]^ environmental‐related applications and energy‐efficient operations,^[^ ^35^ ^]^ and robotics for exploration at extremely harsh conditions.^[^ ^5^, ^36^ ^]^

## Conflict of Interest

The authors declare no conflict of interest.

## Supporting information

Supporting Information

Supplemental Movie 1

Supplemental Movie 2

Supplemental Movie 3

Supplemental Movie 4

Supplemental Movie 5

Supplemental Movie 6

Supplemental Movie 7

Supplemental Movie 8

## Data Availability

The data that supports the findings of this study are available in the supplementary material of this article.
