# Supplementary material for: Exploiting Molecular Dynamics in Composite Coatings to Design Robust Super‐Repellent Surfaces
Source: Adv Sci (Weinh). 2022 Jan 7;9(6):2104331. doi: 10.1002/advs.202104331 (PMC8867138; doi:10.1002/advs.202104331)
Supplement: Supplementary file 1 — Supporting Information [file ADVS-9-2104331-s004.pdf]

ADVANCED SCIENCE
Open Access

Supporting Information

for *Adv. Sci.,* DOI: 10.1002/advs.202104331

Exploiting Molecular Dynamics in Composite Coatings to Design Robust Super-Repellent Surfaces

*Rui Guo, Eirini Goudeli, Wanjun Xu, Joseph J. Richardson, Weijian Xu, and Shuaijun Pan**

Supporting Information

Exploiting Molecular Dynamics in Composite Coatings to Design Robust Super-Repellent Surfaces

*Rui Guo, Eirini Goudeli, Wanjun Xu, Joseph J. Richardson, Weijian Xu, and Shuaijun Pan**

Table of Contents

Materials and Methods S3
Materials S3
Synthesis of POSS S3
POSS–binder solutions S4
Water–ethanol mixtures S4
Finer coatings S4
Coarser coatings S4
Molecular dynamics simulations S5
Surface energy S6
Dynamic contact angles S7
Equilibrium contact angles S7
Roll-off angles S8
Annealing treatment S8
AFM adhesion forces S8
Droplet bouncing dynamics S9
Surface tension S9
Cargo loading S9
Locomotion S10
Breakthrough pressure S10
Solvent stability S11
Peeling test S11
Chemical shielding S11
Aging test S11
Fluorescence test S12
Characterization S12
Statistical analysis S13
Supporting Figure S1–S46 .... S14
Captions for Movie S1–S8 .... S41
Supporting Reference S1–S14 .... S43
Author Contributions S43

Materials and Methods

Materials

Ethyl 2-cyanoacrylate (ECA), sodium hydroxide (NaOH, ≥97.0%, pellet), potassium hydroxide (KOH, anhydrous, ≥99.95%), *n*-hexadecane, ethanol (anhydrous, ≥99.5%), dimethylformamide (DMF), ethylene glycol, and fluorescein isothiocyanate (FITC) were purchased from Sigma-Aldrich. 2,2-Dichloro-1,1,1,3,3-pentafluoropropane was supplied by BOC Sciences. Rapeseed oil and honey were purchased from a local supermarket. Hydrochloric acid (HCl, 32 wt.%) and sulfuric acid (H_2SO_4, 98 wt.%) were purchased from Thermo Fisher Scientific. 1H,1H,2H,2H-Heptadecafluorodecyltriethoxysilane was purchased from Gelest, Inc. The liquid chemicals were used directly as received. Milli-Q water was obtained from an inline Millipore RiOs/Origin water purification system. Muscovite mica (diameter 12 mm) grade V-1 (WSL) was purchased from ProSciTech. Plain silicon wafers (<100> orientation, n-type) were purchased from MMRC Pty. Ltd. and sectioned into 10 mm × 10 mm for use. Mica substrates were mainly used throughout the work for finer coatings unless specified otherwise. Stainless steel meshes (40 × 40, 50 × 50, 70 × 70, 200 × 200, 300 × 300, 400 × 400, 500 × 500, i.e. number of openings per 1 × 1 in^2), stainless steel rod (diameters 500 μm, 1 mm), and copper meshes (60 × 60, 80 × 80, 100 × 100) were supplied by McMaster-Carr. Stainless steel needles (inner diameters: 660, 355, and 203 μm) were purchased from Darwin Microfluidics. Note that stainless steel wires, needles, and mesh substrates were cleaned with ethanol for 10 min, rinsed with copious amounts of ethanol, and dried under nitrogen before use. Tap300 cantilevers (40 N m^{-1}) were supplied by Budget Sensors for AFM imaging. Mesh substrates were used for coarser coatings although copper mesh (100 × 100) was mainly used throughout the work unless specified otherwise. Colloidal probes (1 μm silica and polystyrene (PS) beads) were purchased from Novascan.

Synthesis of POSS

Fluorinated polyhedral oligomeric silsesquioxanes (POSS) was synthesized according to a procedure reported in the literature.[S1,S2] Briefly, 1H,1H,2H,2H-heptadecafluorodecyltriethoxysilane (10 mmol), Milli-Q water (15 mmol), and KOH (37 μmol) were added to a 25 mL round bottom flask containing 10 mL anhydrous ethanol in the protection of nitrogen. The mixture was stirred at 20°C for 17 h. The white precipitate was collected, washed with copious ethanol, and dried in a desiccator under vacuum for >24 h to obtain the final product (white powder, yield: 80–90%).

POSS–binder solutions

POSS–binder solutions (20 mg mL^{-1}) were freshly made (by vortex) by mixing desired amount of POSS and ECA in a fluorinated solvent (i.e., 2,2-dichloro-1,1,1,3,3-pentafluoropropane). A series of POSS–binder solutions were prepared for studying the surface energy of their composites. The concentrations of POSS were 0, 5, 10, 15, 20, 30, 40, 50, 75, and 100 wt.% POSS, respectively. Unless specified otherwise, the 20 wt.% POSS system was mainly used throughout this work.

Water–ethanol mixtures

Different liquids were used to investigate the surface wettability, whereas water–ethanol mixtures (covering a wide range of liquid surface tensions, i.e., 21–72 mN m^{-1}) were mainly used throughout this work unless specified otherwise. Desired volumes of Milli-Q water and anhydrous ethanol were mixed by vortex to obtain the following mixtures: 0, 5, 10, 20, 30, 40, 50, 60, 70, 80, 90, and 100 vol.% ethanol. The water–ethanol mixtures were used for contact angle measurements, roll-off angle measurements, loading capacity experiments, and breakthrough pressure measurements.

Finer coatings

We used drop-casting method to prepare coatings with finer-scale features (i.e., minimizing the effects of substrate roughness and macroscopic textures) to study the intrinsic interfacial properties of POSS–binder systems. Mica substrates (12 mm in diameter) were mainly used as the smooth substrate throughout this work unless specified otherwise. A volume of ~20 μL POSS–binder solutions was drop cast on the mica substrates, followed by polymerization for 24 h at ambient conditions (20°C, humidity 40–70%).

Coarser coatings

Super-repellency was achieved by spraying 20 mg mL^{-1} POSS–binder solutions (20 wt.% POSS) onto mesh substrates (or metal wires). using a Paasche airbrush. The distance between the airbrush nozzle and the substrate was about 20 cm. The spray was driven by compressed air (outlet pressure ~58 psi). After the desired period of spraying time (0–20 min), the coated substrates were kept at ambient conditions for 24 h (20°C, humidity 40–70%) to allow complete polymerization of ECA, followed by the removal of the trapped solvent in a vacuum desiccator for 24 h. A spray time of 20 min (roughly 10 min for each side of the mesh substrate) was mainly used throughout this work unless specified otherwise. Prior to characterizations, the coated super-repellent meshes were blown by compressed air to remove any unbound particles on the coating. For the preparation of super-repellent rafts and boats, a piece of copper mesh (100 × 100) was first cut into desired sizes (e.g., 2.54 cm in diameter) and the

above spray coating steps were applied. In the cargo carrier demonstrations, the fuel container was first glued to the center of the bare mesh surface (followed by spray coating) to ensure the same balanced center of gravity of the system (i.e., less error between measurements).

Molecular dynamics simulations

All-atom molecular dynamics (MD) simulations were performed to study the assembly mechanism, pore size distributions, and solvent stability of POSS–binder composite systems. A binder molecule (i.e., pECA) consisted of a repeating unit n = 50 of monomer ECA. For the assembly simulations, three systems were performed in the NVT (constant number of atoms, volume, and temperature) ensemble at 25°C in a cubic simulation cell of 70 × 70 × 70 $Å^3$ with periodic boundary conditions, including 5 POSS molecules and 5×, 10×, or 15× pECA macromolecules, respectively. For comparing pore sizes, the above 3 systems were simulated at a constant density of 0.5 g mL^{-1} (comparable to the experiments). For solvent stability evaluations, solvent molecules (water, ethanol, or *n*-decane representing solvents with different physiochemical properties, i.e., inorganic vs organic, polar vs nonpolar, high vs low surface tensions) were used. The number of solvent molecules was either fixed at 200 molecules or 6400 atoms (i.e., 200 *n*-decane, 711 ethanol, or 2133 water molecules). The initial configurations were generated using the Materials and Processes Simulations (MAPS) 4.3 platform.[S3] All simulations were performed on the High-Performance Computing cluster[S4] at the University of Melbourne using the large-scale atomic/molecular massively parallel simulator (LAMMPS).[S5] The equations of atomic motion were integrated using the velocity–Verlet algorithm[S6] at a timestep of 1 fs. The polymer consistent force field (PCFF)[S7] was employed with partial charges assigned to each atom based on the bond increments method. The long-range Coulombic interactions were computed using the particle–particle and particle–mesh solver.[S8] The composite systems were simulated for 2−5 ns unless specified otherwise.

The structural configurations of the POSS–binder composites were presented by the radial distribution functions (RDFs), $g(r)$, averaged over the entire simulation cell after the network reached equilibrium. In RDFs, the distance r is the radius of a volume slice from a reference atom or point. Similar calculations are carried out for another reference point and repeated until all referential points in the cell have been used and these results are averaged. Specifically, the POSS–binder $g(r)$ is calculated by counting the number of atoms belonging to the polymer from the center of mass (CoM) of POSS; the POSS–POSS $g(r)$ is determined by counting the number of atoms belonging to POSS from the CoM of another POSS; the fluorine–POSS $g(r)$ is between the CoM of POSS and the 5th carbon atom of the backbone of the fluorinated alkyl group (i.e., roughly the CoM for that chain); fluorine–binder $g(r)$ is radial distance between the 5th carbon atom of the backbone of the fluorinated alkyl group and the entire polymer chain (any atom belonging to the polymer); and the binder–binder $g(r)$ represents both the intra- and inter-molecular radial distances.

The pore size distributions were obtained using the software package Zeo++[S9] implemented in MAPS using different probe radii (0.5 Å and 2.8 Å). Noted that the calculated pore size distributions was dependent on the initial MD conditions, for example, the probe size, the numbers of solvent molecules/atoms, the density of composite in the system, and the POSS/binder ratio, which were all investigated in this work. The pore sizes are the average of 2000 measurements performed at a radial distance step of 0.05 Å in the range of 0–70 Å. The networks were visualized using visual molecular dynamics (VMD).[S10] The mass distributions were exported as heatmaps. The relevant movies were exported and re-recorded (to compress) using an online tool (Screencast-O-Matic).

The interaction energies between POSS and binder, POSS and solvent, and binder and solvent were determined in LAMMPS[S5] at 25°C for the composite system containing 5 × POSS and 5 × pECA (n = 50) in a cubic simulation cell of 70 × 70 × 70 $Å^3$ with periodic boundary conditions. The number of solvents was fixed at 6400 atoms (i.e., 200 *n*-decane, 711 ethanol, or 2133 water molecules). Three independent simulations for each system were carried out for 2–6 ns. The average interaction energy (and the standard deviation) between POSS and binder was −184.5 ± 12.5, −293.6 ± 20.9, and −390.5 ± 16.4 kcal mol^{-1} in ethanol, water, and *n*-decane, respectively. The POSS–binder contact area was derived based on the solvent accessible surface area (SASA) of the POSS molecules, the binder molecules, and the POSS–binder aggregates. The SASA was calculated using a solvent probe radius of 1.4 Å using a TCL script written for VMD.[S10] The temporal evolution of the POSS–binder contact area was determined using one MD simulations for at least 2.5 ns. The contact area between the POSS and the binder was about 35, 40, and 50 nm^2 in ethanol, water, and *n*-decane, respectively.

Surface energy

Solid surface energy consists of the polar (γ_s^p, dipole-hydrogen bonding) component and the nonpolar (γ_s^d, dispersive) component. The Owens–Wendt method[S11] was adopted for determining both components of the surface energy of POSS–binder composites.

$$\frac{1+\cos\theta_a}{4} = \sqrt{\gamma_s^d \gamma_l^d} + \sqrt{\gamma_s^p \gamma_l^p} \qquad \text{(S1)}$$

where θ_a, γ_l^d, and γ_l^p are advancing contact angle, dispersive, and polar components of the probe liquid. A polar liquid (i.e., water) and a nonpolar liquid (i.e., *n*-hexadecane) were used as the probe liquids. The polar and nonpolar components of water's surface tension are 51.0 and 21.1 mN m^{-1}, respectively, while *n*-hexadecane only has a nonpolar component (i.e., 27.5 mN m^{-1}). Knowing their contact angles on the finer coatings of POSS–binder systems, both surface energy components and the total surface energy ($\gamma_s^p + \gamma_s^p$) were computed by the above equation. Multiple measurements (>3) were carried out to give average values, and the standard deviations were reported in this work. It is noted that the surface energy

was calculated using advancing contact angles which is larger than the equilibrium contact angle (if any), therefore, the true surface energy should be slightly higher than the calculated.

Dynamic contact angles

Dynamic contact angles (advancing contact angle, receding contact angle) were measured both on the finer coatings and the coarser coatings. The contact angles on the finer coatings represent the intrinsic wettability of the POSS–binder composite (or the local wettability for coarser surfaces), while those on the coarser coatings are the apparent contact angles reflecting a combined effect of the intrinsic characteristics of the coating system (i.e., surface chemistry), the roughness, and re-entrant texture of the surface (i.e., structural effect). A tensiometer (DataPhysics OCA 20) was used to measure the contact angles. A volume of ~5 μL liquid was pumped onto the coating surface with the needle (outer diameter 0.46 mm) kept immersed. By further increasing the liquid volume (e.g., up to 10 μL) or decreasing the volume (e.g., until most of the liquid was taken away by the needle), the advancing (largest value) or receding (smallest) contact angles were determined. Experiments were repeated at least 3 times for each sample, and the average values and standard deviations were reported in this work. Contact angle hysteresis (CAH) was calculated by comparing the difference between the advancing and receding contact angles. Larger CAHs indicated stronger interactions between the probe liquid and the solid surface, while smaller CAHs were a sign of low interfacial interactions.

Equilibrium contact angles

As the finer coatings investigated were not ideally smooth and thereby one could not directly measure the equilibrium contact angles but could determine through calculations using the corresponding dynamic contact angles:[S12]

$$\theta_{\mathrm{e}} = \arccos\left(\frac{k_{\mathrm{a}}\cos\theta_{\mathrm{a}} + k_{\mathrm{r}}\cos\theta_{\mathrm{r}}}{k_{\mathrm{a}} + k_{\mathrm{r}}}\right) \qquad \text{(S2)}$$

where θ_{a} and θ_{r} are the advancing and receding contact angles and the coefficients can be computed below:

$$k_{\mathrm{a}} = \left(\frac{\sin^3\theta_{\mathrm{a}}}{2 - 3\cos\theta_{\mathrm{a}} + \cos^3\theta_{\mathrm{a}}}\right)^{1/3} \qquad \text{(S3)}$$

$$k_{\mathrm{r}} = \left(\frac{\sin^3\theta_{\mathrm{r}}}{2 - 3\cos\theta_{\mathrm{r}} + \cos^3\theta_{\mathrm{r}}}\right)^{1/3} \qquad \text{(S4)}$$

Roll-off angles

Roll-off angles were determined both experimentally and computationally in this work. For measurements, a droplet of liquid (~10 μL) was first gently placed on the sample surface which was horizontally placed on the stage of the tensiometer (DataPhysics OCA 20). The angle of the stage was then manually tilted with a gradual increase until the droplet rolled off the surface. The minimum tilt angle required for the droplet rolling off the surface was recorded as the roll-off angle. The measurements were repeated >3 times, and both average values and their standard deviations were reported in this work. For theoretical analysis, roll-off angles can be predicted from the diameter of the triple-phase contact line (d):[S13]

$$d = 2\sin\bar{\theta}_{\mathrm{e}}^{*}\sqrt[3]{\frac{3V}{\pi\left(2-3\cos\bar{\theta}_{\mathrm{e}}^{*}+\cos\bar{\theta}_{\mathrm{e}}^{*}\right)}} \quad \text{(S5)}$$

where V is the volume of the droplet. $\bar{\theta}_{\mathrm{e}}^{*}$ is the average equilibrium contact angle (the star symbol is to indicate the (coarser scale) super-repellent surface), which can be computed using Equation S2–S4.

Annealing treatment

Annealing treatment of POSS–mica surfaces was carried out in this work to obtain a relatively smoother (than non-annealed) surface for AFM force measurement. POSS–coated mica was placed in a chamber furnace (Shimaden FP21 controller), and the temperature was increased from room temperature (20°C) to 200°C at a ramp rate of 10°C min^{-1}. The sample was kept at 200°C for 30 min followed by (natural) cooling to room temperature. The cooling took about 4 h. The gas medium was ambient air. Both amorphous and crystalline regions, formed after the annealing treatment, were subjected to AFM force measurements. Other finer coating samples were also treated in the same way as controls.

AFM adhesion forces

Surface adhesion forces were examined in the air using the colloidal-probe AFM technique. Both hydrophobic (PS) and hydrophilic (silica) colloidal probes were used to evaluate the adhesive property of the polymeric binder as well as the non-adhesive property of POSS. The spring constants of the colloidal probes (1 μm SiO_2 or PS bead, Novascan) had a nominal spring constant of 16 N m^{-1}, which was calibrated before use (in the range of 26–29 N m^{-1}). The calibration and adhesion force measurements were performed using Cypher AFM (Asylum Research) instrument. Trigger point, force distance, and scanning rate were 1–2 μN, 200 nm, and 0.5 Hz, respectively. A total number of 100 measurements for each sample was carried out at different locations. The Gaussian distribution, average force, and standard deviations were reported in this work.

Droplet bouncing dynamics

Ethanol bouncing dynamics were recorded using a high-speed camera (Fastec HiSpec1) at a frame rate of 3900 fps. The ethanol droplet (diameter ~2.4 mm) was released at a height of ~7 mm above the super-repellent surface. The bouncing trajectory, contact time, and deformation (both in horizontal and vertical directions) were analyzed using FASTCAM Viewer 4 (PFV4).

Surface tension

We used the pendant drop method to determine the surface tension of water-ethanol mixtures. A tensiometer (DataPhysics OCA 20) was used and experiments were carried out at room temperature (20°C). A volume of ~7 μL liquid was pendant by a stainless-steel syringe needle (outer diameter 0.46 mm), avoiding shaking or other turbulences. The shape of the pendent drop was tracked by its in-built camera at a frame rate of 10 fps and analyzed. One measurement was completed once the liquid surface tension became constant, which usually took 2–10 min. At least 3 measurements were performed, and both average surface tensions and their standard deviations were reported in this work.

Surface tension measurements were also carried out to assess if chemical leaching occurred for samples subjected to different stability tests (e.g., immersion in solvents, peeling test, heat treatment, chemical shielding test, etc.). These samples (5 mm x 10 mm) were immersed in 1 mL water for 10 min. After removing the substrate, the water left was subject to surface tension measurement to see if its surface tension changed. Decreased or increased values indicated chemicals were leached either from the coating or due to the solvent residue left from the previous immersion test. For example, as a control, a non-coated mesh substrate was first immersed in ethanol for 10 min. Then it was transferred to Milli-Q water and kept for another 10 min. The water remaining showed a much-reduced average surface tension (67 mN m^{-1}), indicating the ethanol residue was transferred to the water causing the drop of its surface tension. In contrast, if no leaching occurred (e.g., our super-repellent surface), the surface tension values were constant even after the surface being immersed in ethanol.

Cargo loading

The loading capacity of our super-repellent surfaces (rafts and boats) was determined by the maximum volume of the liquid added to the container mounted on the super-repellent surfaces. The load capacity was the total weight of the surface (raft 0.385 ± 0.002 g; boat 0.448 ± 0.002 g), the container (~1 g), and the weight of the liquid loaded (e.g., water). The weight of the liquid loaded was converted from its volume added. For each loading case (i.e., on the surface of water–ethanol mixtures), at least 3 different measurements were repeated, and both the average values and their standard deviations were reported in this work. Note, during the measurement, adding water (as the weight) should be continuous and slow

to avoid turbulence to the water–ethanol surface, i.e., minimizing the measurement error due to the contact instability (low friction) between the coating and surface of the pool liquid.

Locomotion

Three different types of locomotion on the surface of water were demonstrated using our super-repellent surface in this work, i.e., pulsive motion, continuous linear motion, and rotational motion. These motions were driven by the surface tension gradients between the front and the rear of the surface. The surface tension gradients were achieved by the low surface tension liquid (ethanol) loaded, which was released at the rear of the raft via a siphon effect through a stainless steel needle bent into a V-shape with one end (end A) immersed in the ethanol container and another (end B) placed at the air–water interface at the rear of the raft. When the end B was immersed in the pool liquid, continuous motions were achieved (linear or rotational). The direction of continuous motion was dependent on the area of the waterway. When a narrow (slightly wider than the raft) waterway was used, the raft moved linearly ahead. Otherwise, if an open waterway was used, a boat floating on it would move rotationally. However, if the end B was suspended slightly on the surface of the water, the raft displayed a pulsive motion instead. Each pulsive motion corresponded to one release of an ethanol droplet into the waterway. The ethanol releasing interval was dependent on the height of the fuel liquid. Note that the speed of the locomotion could be tuned by the surface tension gradient generated, e.g., using low surface tension fuel liquid, using a needle with a wider inner diameter (i.e., more/quicker liquid releasing).

In addition, we compared the motion of super-repellent rafts on various liquids including viscous liquids, such as ethylene glycol (16 mPa·s, 20°C), rapeseed oil (70 mPa·s, 20°C), honey (~10^4 mPa·s, 20°C). Note the viscosity of water is 1 mPa·s. The surface tensions (at 20°C) are 34, 48, 62, and 72 mN m^{-1} for rapeseed oil, ethylene glycol, honey, and water, respectively. These liquids (5 mL) were first added to glass petri dishes (diameter 5 cm) serving as the liquid pathway. The rafts (5 mm × 5 mm) (i.e., the coated copper meshes (mesh size 100 × 100)) were placed on these liquids. A drop of ethanol (~10 µL) was then added near the raft. The moving distance of the raft was recorded and analyzed. It is noted that this experiment was only to further explore the interfacial property of the super-repellent coatings.

Breakthrough pressure

Liquid breakthrough pressures (P_b) of the super-repellent mesh surfaces were determined by a customized apparatus. A piece of the coated mesh was sandwiched between two columns and placed vertically, with a large container placed under it to collect the permeate. The testing liquid (water–ethanol mixtures) was added slowly into the upper column until the liquid started to pass through the mesh. The corresponding breakthrough pressure was calculated using the liquid height (h) and density (ρ), i.e., $P_b = \rho g h$. At least three measurements were carried out for each testing liquid, and the average

values with standard deviations were reported in this work. The measured values were compared with the theoretical predictions as well.

Solvent stability

In addition to the MD simulations with added solvent molecules, the super-repellent coatings' solvent stability was also evaluated experimentally. The coated mesh substrates were immersed for 10 min in water, ethanol, *n*-decane, DMF, 98 wt.% H_2SO_4, or ~19 M NaOH, respectively. Briefly, saturated NaOH aqueous solution (~19 M) was prepared by adding excessive NaOH pallets (~20 g) to Milli-Q water (10 mL) in a glass beaker. The mixture was stirred for ~10 min, followed by sedimentation and cooling to room temperature for ~30 min. The supernatant was used as the saturated NaOH in this work without further treatment. *Caution! Concentrated H_2SO_4 and NaOH are highly corrosive and can cause violent reactions and damage to skin and clothes and should be handled very carefully in a fume hood with suitable personal protective equipment.* After immersion, the substrates were transferred to 1 mL water and kept immersed for another 10 min. The used water was subjected to surface tension measurement to confirm if any chemicals were leached from the coating or transferred from the first immersion step.

Peeling test

The mechanical durability of the super-repellent coatings was tested by peeling test. The coated mesh was placed on the benchtop, and a strip of transparent 3M tape was applied to the coating by an external weight of 1 Kg. The load was kept for 5 min, after which the load was removed, and the tape was peeled off. The roll-off angles of water–ethanol mixtures and solvent stability (i.e., immersion stability) were tested.

Chemical shielding

We use highly corrosive chemicals to demonstrate the chemical shielding property of the super-repellent coatings. The corrosive liquids used in this work included 98 wt.% H_2SO_4, 32 wt.% HCl, and ~19 M NaOH. *Caution! These liquids are highly corrosive and should be handled very carefully in a fume hood with suitable personal protective equipment.* Chemical shielding was demonstrated by the abovementioned solvent stability test as well as the rolling droplet test. As for the rolling droplet test, these liquids were released from a height of 10–20 mm above the substrate (tilt angle 5–10°). After impingements, the droplets rolled off the coatings with no stains or damages left.

Aging test

Aging tests adopted in this work included plasma treatment and UV–O_3 treatment. As for UV–O_3 treatment, the super-repellent coatings were placed in the drawer of an Ultraviolet Radiator (BioForce Nanosciences, model: UV.TC.EU.003) and treated for 20 min. *Warning! The drawer must be closed to operate to avoid UV irradiation to the operator.* As for the air plasma treatment, a Harrick Plasma Cleaner was used, and the sample was placed in the chamber and treated on high (RF level) for 60 s while maintaining a chamber pressure of 500 bar.

<u>Fluorescence test</u>

We prepared 1 mg mL^{-1} fluorescein isothiocyanate (FITC) in ethanol as the staining solution. The coated mesh substrate (5 mm × 10 mm) was kept immersed in the solution for 10 min. A piece of non-coated mesh substrate were used as the control substrate. After immersion test, the mesh substrates were imaged using a fluorescent microscope. The excitation laser source was 488 nm spectral line. Where there was fluorescence detected, we considered the surface being contaminated by the staining solution.

<u>Characterization</u>

The surface morphology of the coatings was characterized by a Hirox RH-2000 2D/3D digital microscope, scanning electron microscopy (SEM) using an FEI Quanta 200 environmental scanning electron microscope with an operating voltage of 5 kV. The surface chemistry was characterized by energy-dispersive X-ray spectroscopy (EDX), and Attenuated Total Reflection Fourier-transform infrared (ATR FTIR; Bruker) spectroscopy. Differential Scanning Calorimeter (Perkin Elmer) was used to determine the melting point of POSS with a heating and cooling rate of 5°C min^{-1} and nitrogen flow rate of 50 mL min^{-1}. The POSS sample (19.440 mg) was heated from 25°C to 200°C, cooled to −80°C, and finally heated to the start temperature (i.e., 1 cycle). An IntraCooler II was used to control the temperatures. The thermal stability (e.g., weight loss) of POSS was characterized by a thermogravimetric analyzer (TGA 8000, PerkinElmer) in the air (20 mL min^{-1}) using heating and cooling rates of 5°C min^{-1}. The sample (~10 mg) was kept at 200°C for 2 h and the weight loss was calculated after the TGA measurement.

Surface roughness of finer coatings was characterized in tapping mode using Cypher AFM (Asylum Research) instrument. Tap300 cantilevers (40 N m^{-1}, Budget Sensors) were used for imaging in the air. The roughness values reported are RMS (calculated as the root-mean-square of the measured microscopic peaks and valleys on the sample surface). At least 5 measurements on different locations (2 × 2 μm^2) roughly from the center to the edge of the coating layer were conducted. The reported values were the average values (and standard deviations). Coating thickness was measured by AFM, where the sample was scratched using a blade. The thickness was thereby determined by comparing the height difference.

Statistical analysis

We used a mass-based (or density-based) normalization for all the MD simulations in this work. For radial distribution functions (RDFs, $g(r)$), data shown was the time-averaged over a period of 0.5 ns to show the variations in the structure, i.e., 1 ns corresponds to the time-average RDFs from 0.75–1.25 ns, 2 ns corresponds to the time-average RDFs from 1.75–2.25 ns. The RDFs graphs were generated from one MD simulations for each system, and the software Origin 2019b was used. The heatmaps describing the mass/aggregates distributions were generated using MATLAB, and a blue-to-yellow (i.e., vacuum-to-mass aggregates) color coding was used to present the mass distribution in the simulation cell. For the pore size distributions, 2000 measurements were performed by MD simulations using a given probe size (with a radius of 2.8 Å or 0.5 Å) searching at a radial distance step of 0.05 Å in the range of 0–70 Å, which was averaged to determine the average pore size.

For the height distributions when calculating the surface roughness, the raw datasets and Gaussian fitting were generated by the built-in analysis tool of the Asylum Research Cypher software. The number of data points depend on the AFM scanning rate and resolution (e.g., 256 × 256 pixels per image). The AFM height was not the absolute values but the normalized by the mean, and a color bar was used to indicate the relative height for each AFM height image. RMS roughness values were calculated using the built-in function of the Cypher AFM software. At least 5 measurements on different locations (2 × 2 μm^2, roughly from the center to the edge of the coating layer) were conducted. Standard deviations were calculated using Microsoft Excel, and the average values (and standard deviations) were exported using Origin 2019b. As for the AFM adhesion force experiments, 100 measurements for each sample was carried out at different locations. The adhesion values were the difference between the initial state (no contact) and the maximum pull-off force, determined by the built-in function of the instrument software. The data was presented as relative frequency, fitted by normal Gaussian function (automatic binning) using Origin 2019b. The average adhesion forces (and standard deviations) of the 100 datasets were calculated using Excel.

Liquid surface tensions were analyzed using at least 10 independent measurements for each system and presented by Violin plot (Origin 2019b). Normal symmetric distribution curve was used and the data was shown as box. Due to the small sample size, the significant differences of these datasets were not assessed. The Violin plot was only to show the range of the surface tensions, and no statistical test was applied.

As for the other datasets regarding surface energy, contact angles, roll-off angles, cargo loading capacity, and breakthrough pressure, we simply used the average function and the standard deviation function of Excel for the statistical analysis. In these experiments, the sample size was at least three (unless specified elsewhere). The errors are the standard deviations.

Supplementary Figure S1–S46

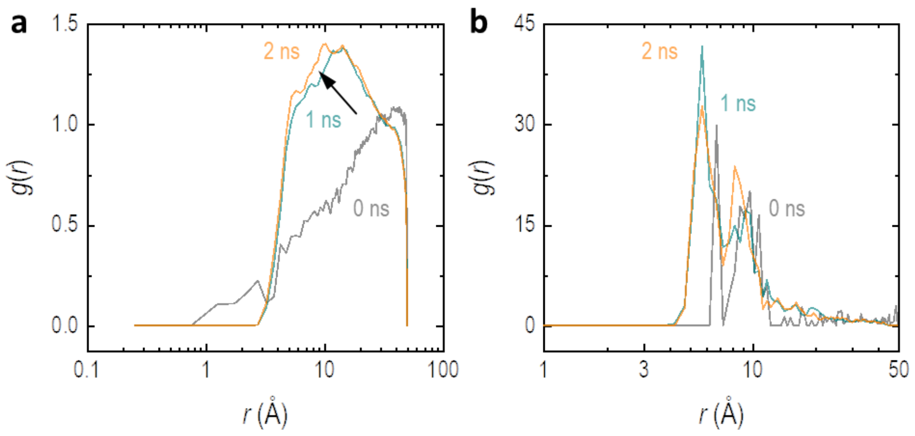

Figure S1. Inter-species RDFs of POSS–binder (5:10) composite by MD simulations. (a) Fluorine–binder $g(r)$ and (b) fluorine–cage (of POSS) $g(r)$ at different simulation times (i.e., 0 ns, 1 ns, and 2 ns), where 0 ns corresponds to the initial configuration.

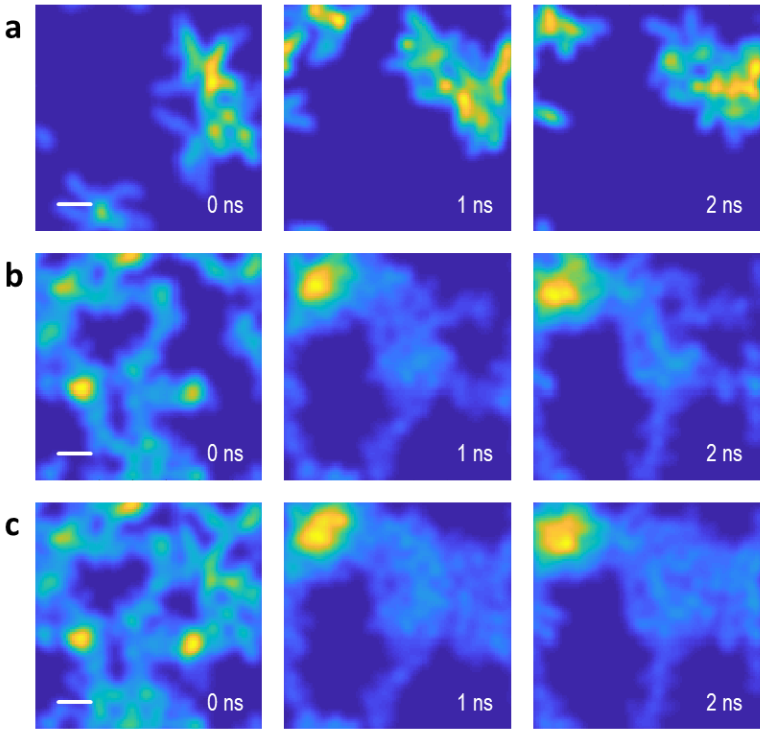

Figure S2. Projected mass distributions in the POSS–binder (5:5) composite by MD simulations. Blue-to-yellow (i.e., vacuum-to-mass aggregates) color coding is used, and the dark blue and light yellow correspond to vacuum and the aggregated mass, respectively: (a) POSS molecules, (b) binder molecules, and (c) POSS–binder composite (i.e., overlapped image of a and b).

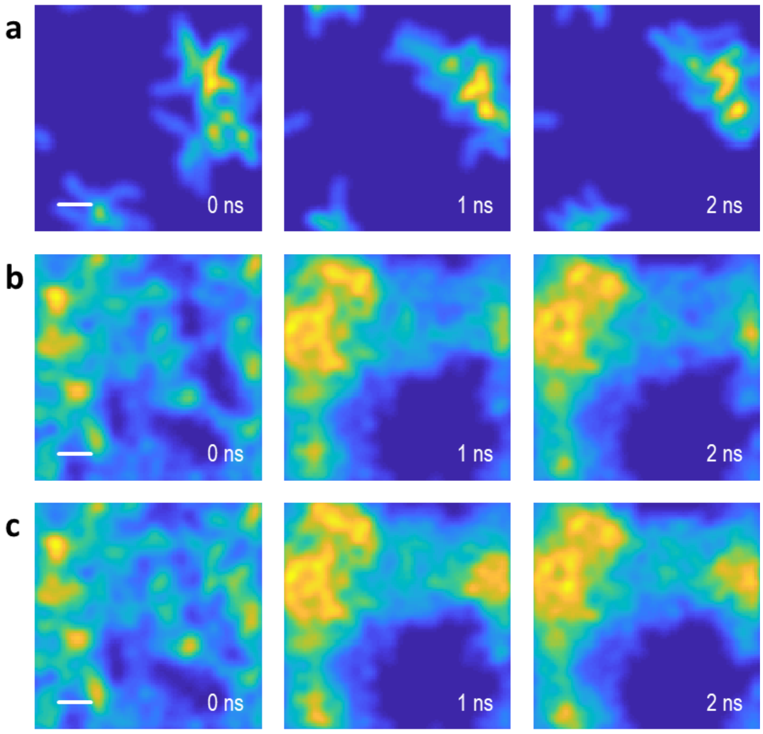

Figure S3. Projected mass distributions in the POSS–binder (5:15) composite by MD simulations. Blue-to-yellow (i.e., vacuum-to-mass aggregates) color coding is used, and the dark blue and light yellow correspond to vacuum and the aggregated mass, respectively: (a) POSS molecules, (b) binder molecules, and (c) POSS–binder composite (i.e., overlapped image of a and b).

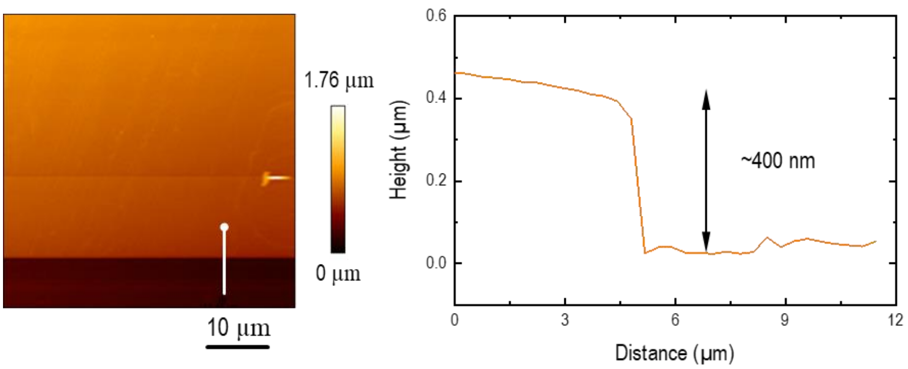

Figure S4. Thickness of the composite POSS-binder coating on silicon wafer. The sample was first scratched using a blade and imaged using AFM. The dark area is the substrate. The thickness of the composite coating was determined by the height difference between the light area (coating) and the dark area (substrate).

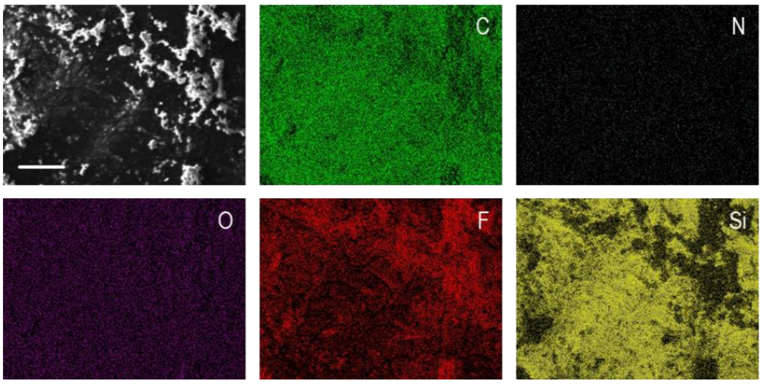

Figure S5. SEM image and EDX mapping of a finer composite coating (20 wt.% POSS). The elements imaged include C, N, O, F, and Si as displayed on the images. Scale bar is 200 μm and applies to all of the images.

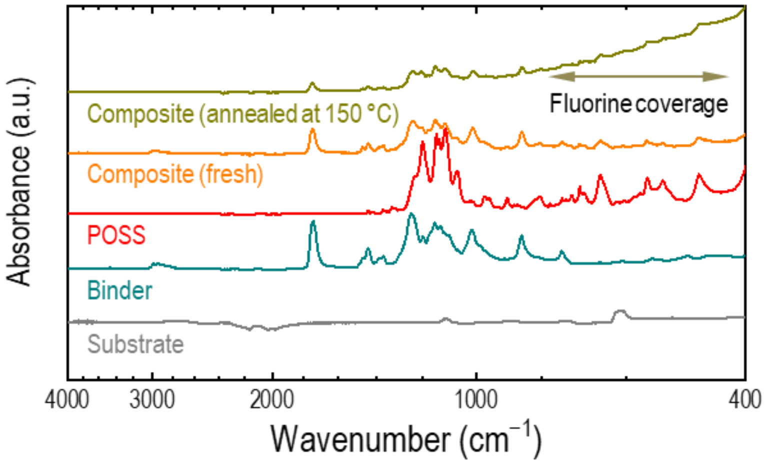

Figure S6. ATR FTIR spectra of finer coatings. The surfaces include the plain substrate (i.e., silicon wafer), pure binder coating (i.e., pECA), pure POSS, and their composite (20 wt.% POSS). When annealed at or above the melting point of POSS (~150°C), the surface fluorine coverage increases, indicated by the increased absorbance at wavenumbers of 400–800 cm^{-1} (i.e., CF_2, CF_2–CF_2, CF_2–CF_3, CF_3).

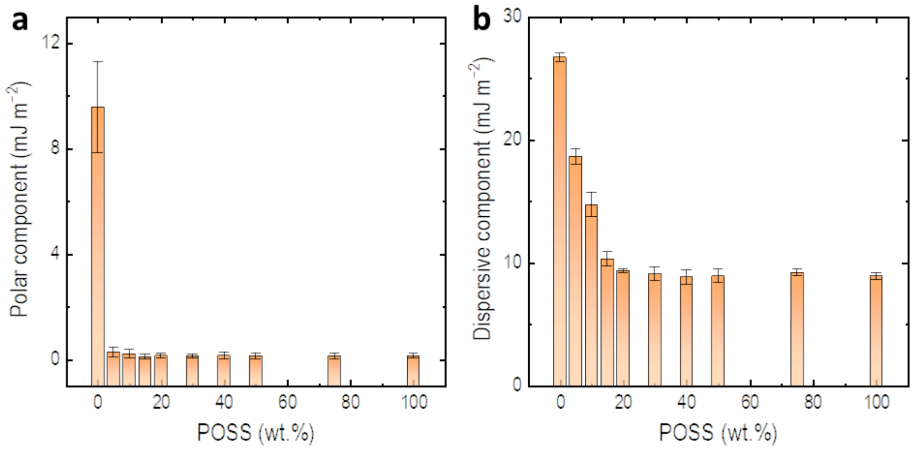

Figure S7. Surface energy components of the POSS–binder composites. (a) Polar (dipole hydrogen-bonding) component. (b) Dispersive (nonpolar) component. The composites are nonpolar in nature with the dispersive component as the dominant in their surface energy components. Errors are standard deviations of >3 measurements.

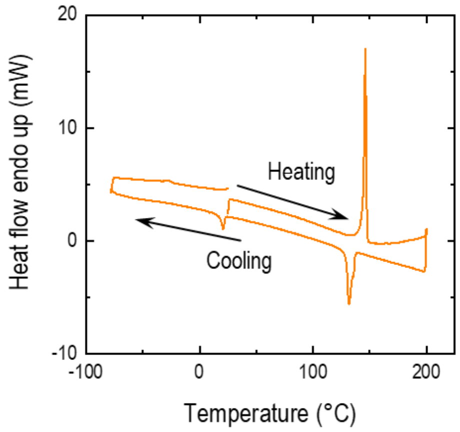

Figure S8. DSC thermogram of POSS. The heating and cooling rates are 5°C min^{-1}. Melting of POSS occurs at around 150°C.

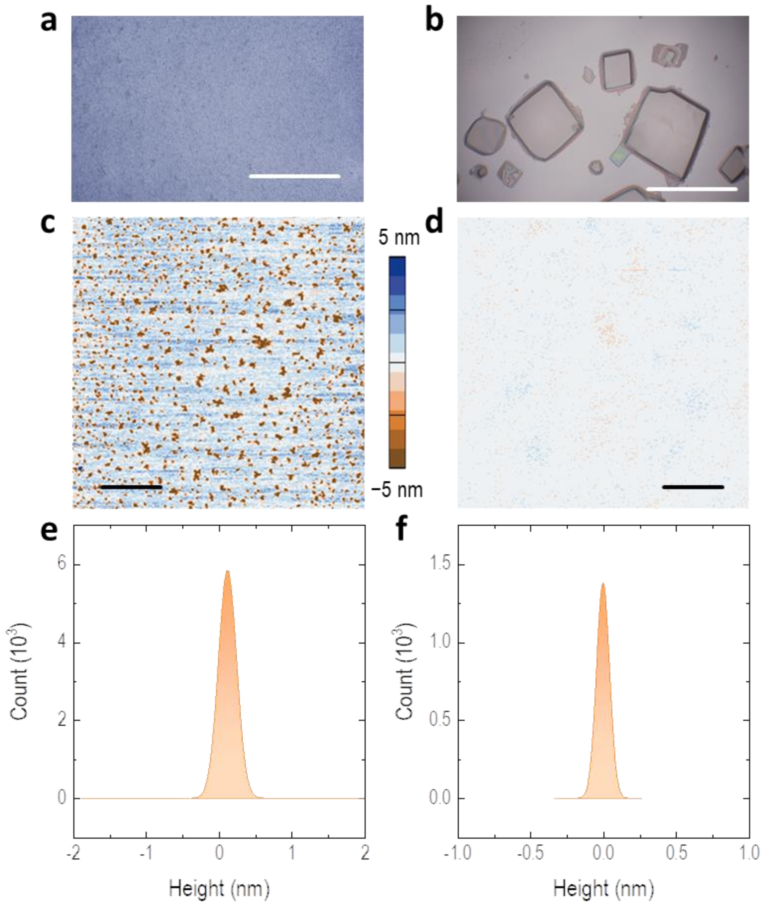

Figure S9. Crystallization of POSS on mica surface. (a) Microscope image of POSS-coated mica surface (non-annealed). Scale bar is 500 μm. (b) Microscope image of the annealed POSS (at 200°C) showing both amorphous and crystalline regions. Scale bar is 200 μm. (c, d) AFM images of a typical amorphous area (c) and crystalline area (d). The color scale applies to both images. Scale bars are 2 μm. (e, f) Height histograms of the amorphous (e) and the crystalline (f) regions, with the RMS roughness (and standard deviation) of 357 ± 15 pm and 44 ± 3 pm, respectively. Sample sizes in (e and f) were >60000, and normal Gaussian distribution function was used.

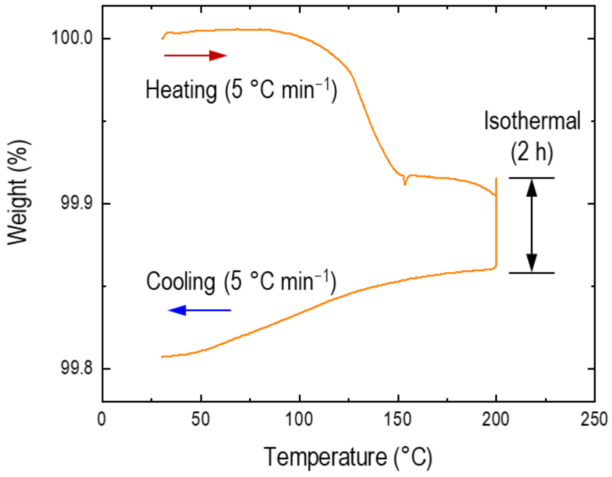

Figure S10. Thermal stability of POSS. The POSS sample (~10 mg) was heated to 200°C and maintained at 200°C for 2 h before cooling to 30°C. The heating and cooling rates were 5°C min^{-1}. The TGA analysis was carried out in the air (20 mL min^{-1}). The weight loss was less than 0.2 wt.%.

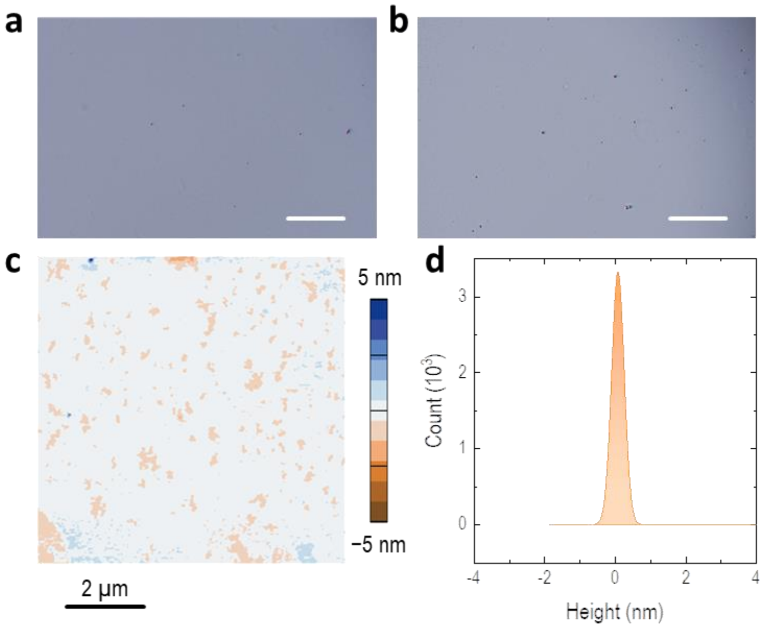

Figure S11. Annealing of the binder surface. (a, b) Microscope images of fresh binder (a, pECA) and annealed (b). Scale bars are 200 μm. (c, d) AFM image of the annealed binder surface (c) and (d) its height histogram. The RMS roughness (and standard deviation) of the fresh and annealed binder surface are 1024 ± 417 pm and 288 ± 49 pm, respectively. Sample size was >60000, and normal Gaussian distribution function was used.

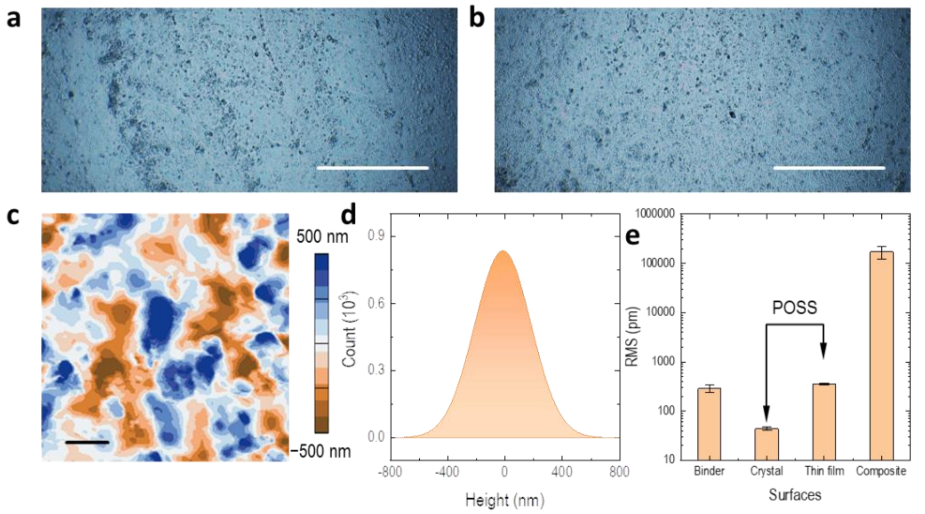

Figure S12. Annealing of the composite (20 wt.% POSS) surface. (a, b) Microscope images of the fresh (a) and the annealed coating (b). Scale bars are 200 µm. (c, d) AFM image of the annealed surface (c) and (d) its height histogram. Sample size was >60000, and normal Gaussian distribution function was used. (e) RMS roughness of the annealed surfaces. Errors are standard deviations of >5 measurements.

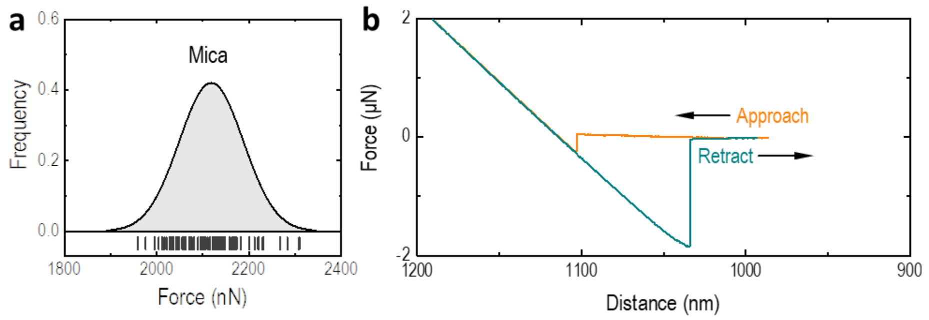

Figure S13. Adhesion forces determined by silica colloidal-probe AFM. Force distribution (a) and representative force–distance curves (b) between the colloidal probe and the surface of the mica substrate.

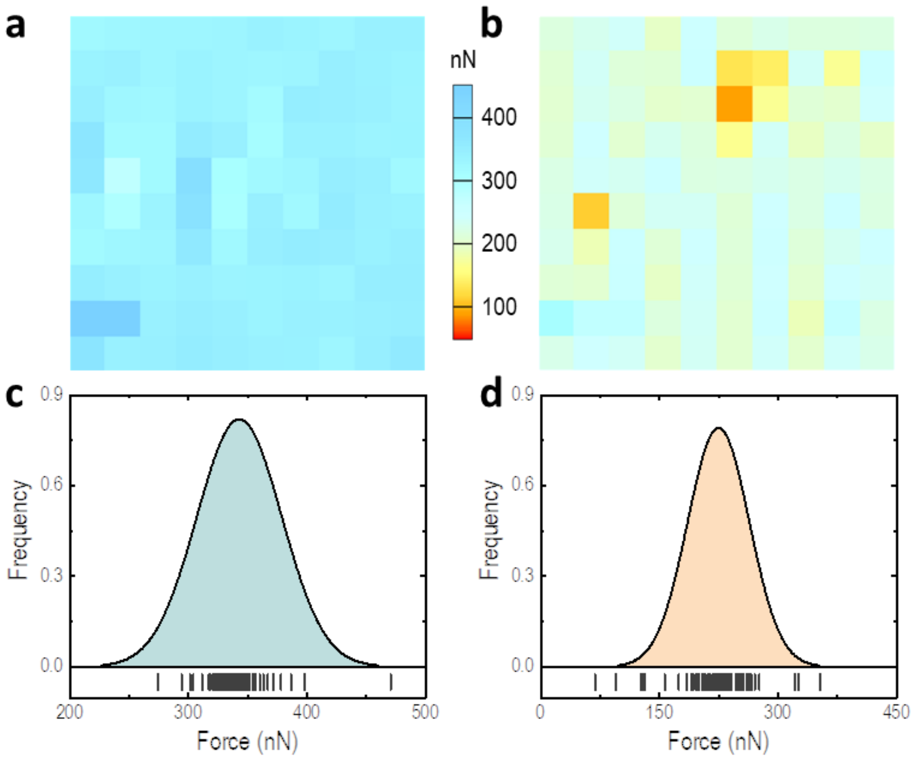

Figure S14. Adhesion forces determined by PS colloidal-probe AFM. (a, c) Heatmap (a) and force distribution (c) of the binder surface (10 × 10 μm^2). (b, d) Heatmap (b) and force distribution (d) of the annealed POSS surface (amorphous area, 10 × 10 μm^2). Sample sizes were 100, and normal Gaussian distribution function was used.

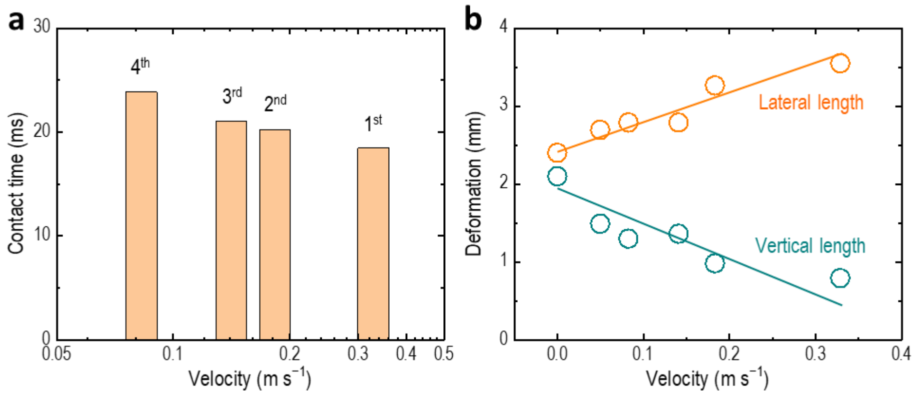

Figure S15. Bouncing dynamics of an ethanol droplet (diameter ~2.4 mm). (a) Contact time of each impact (different impact velocities) during the measurement. (b) The maximum deformation (diameter or length) of the ethanol droplet at the lateral and the vertical directions during each impact.

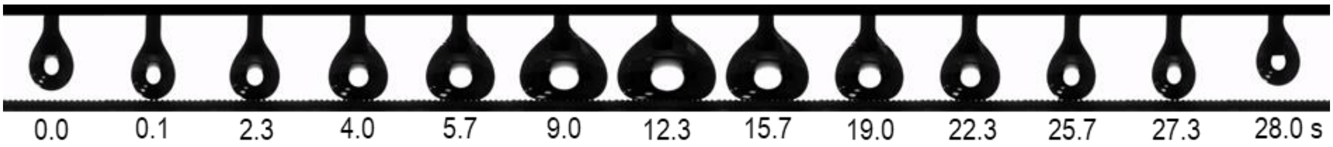

Figure S16. Ultralow CAH of the super-repellent mesh surface. The probe ethanol droplet was advancing and receding on the surface displaying negligible hysteresis. The images were recorded by the built-in camera of the tensiometer. The initial droplet volume was about 5 μL.

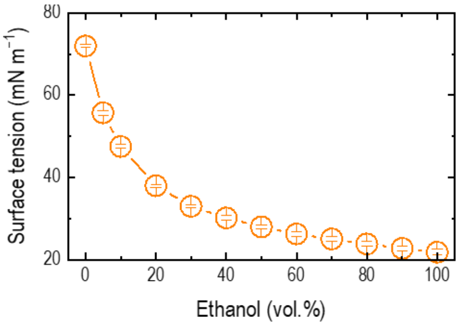

Figure S17. Surface tension of water–ethanol mixtures at 20°C. The errors are standard deviations of >3 measurements.

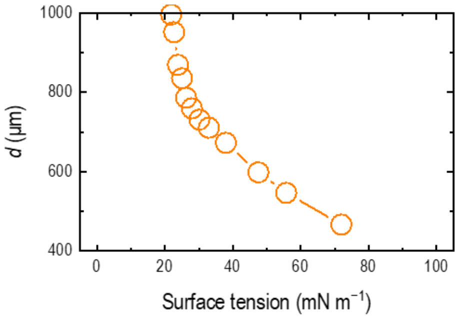

Figure S18. Diameter of triple-phase contact line (*d*) on a super-repellent copper mesh surface (100 × 100). The liquids are water–ethanol mixtures. The values of *d* were computed using Equation S6.

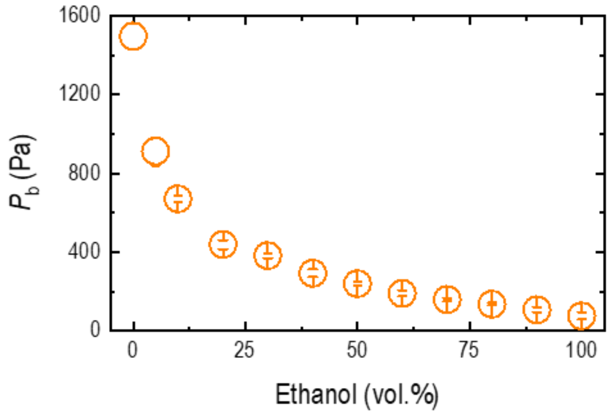

Figure S19. Measured breakthrough pressure of water–ethanol mixtures. The 100 × 100 copper mesh coated with POSS–binder was used as the substrate. Errors are standard deviations of >3 measurements.

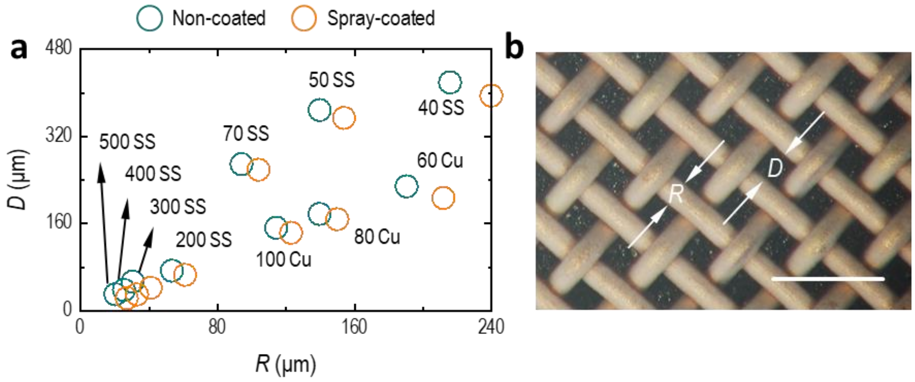

Figure S20. Mesh substrates. (a) Wire and inter-fiber diameters of the mesh substrates before and after 20 min coating. (b) The microscope image of spray-coated super-repellent copper mesh (100 Cu). Scale bar is 500 μm. SS is for stainless steel and Cu is for copper. The numbers, e.g., 100 Cu, indicate the size of the mesh, i.e., the number of parallel wires in 1 in.

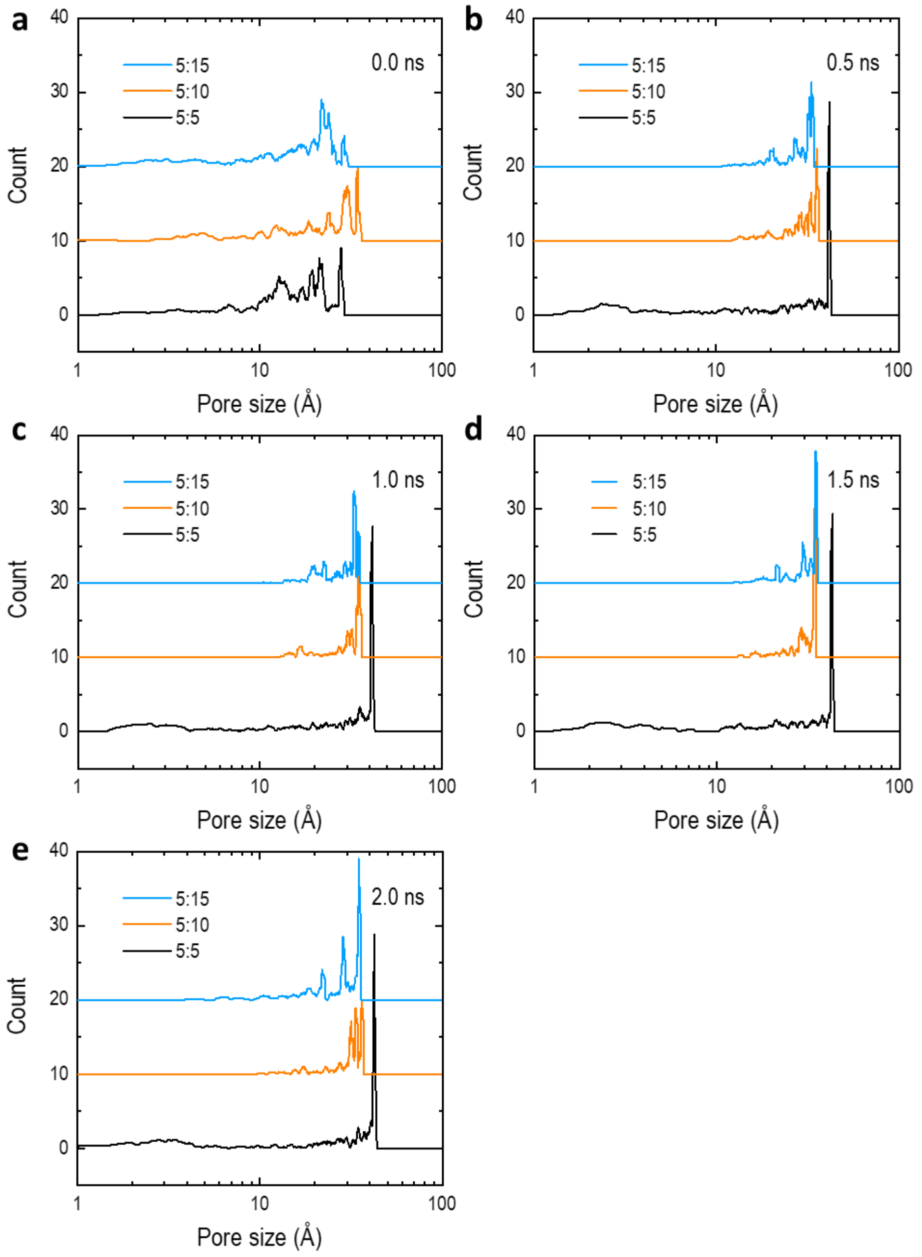

Figure S21. Pore size distributions in POSS–binder systems (probe radius 0.5 Å). (a–e) Pore sizes at different simulation times, i.e., 0–2 ns. Three systems are 5:5, 5:10, and 5:15 POSS–binder composites, respectively.

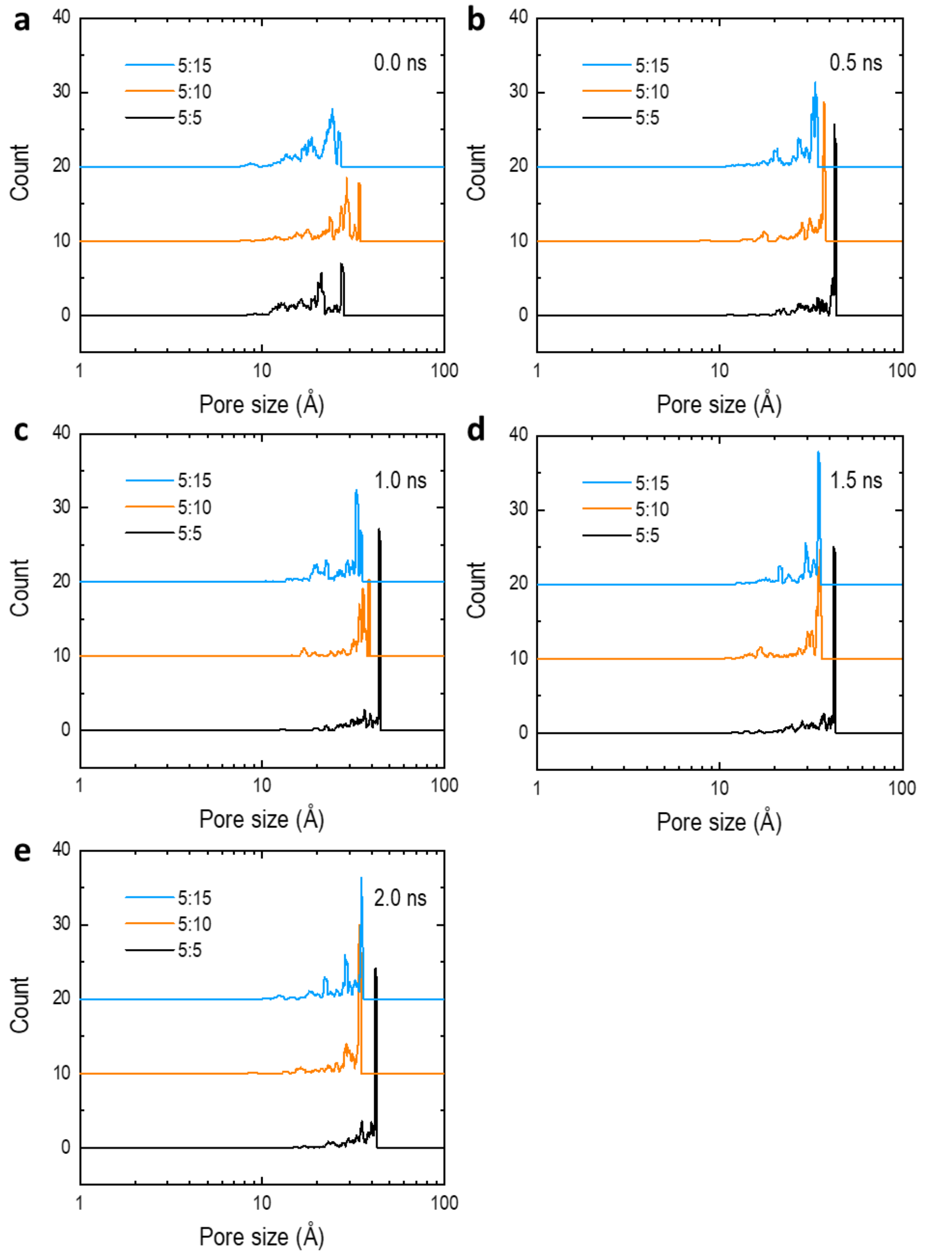

Figure S22. Pore size distributions in POSS–binder systems (probe radius 2.8 Å). (a–e) Pore sizes at different simulation times, i.e., 0–2 ns. Three systems are 5:5, 5:10, and 5:15 POSS–binder composites, respectively.

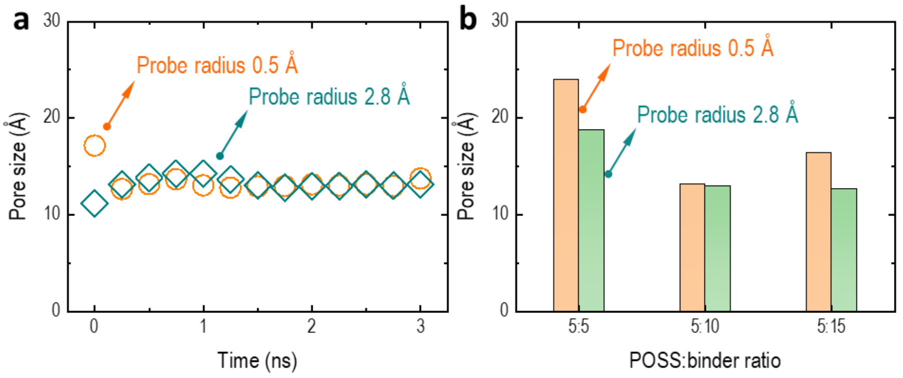

Figure S23. Comparison of pore size simulations. (a) The evolution of average pore sizes as a function of simulation time when using different probe radii. (b) Comparison of average pore sizes in different POSS–binder systems.

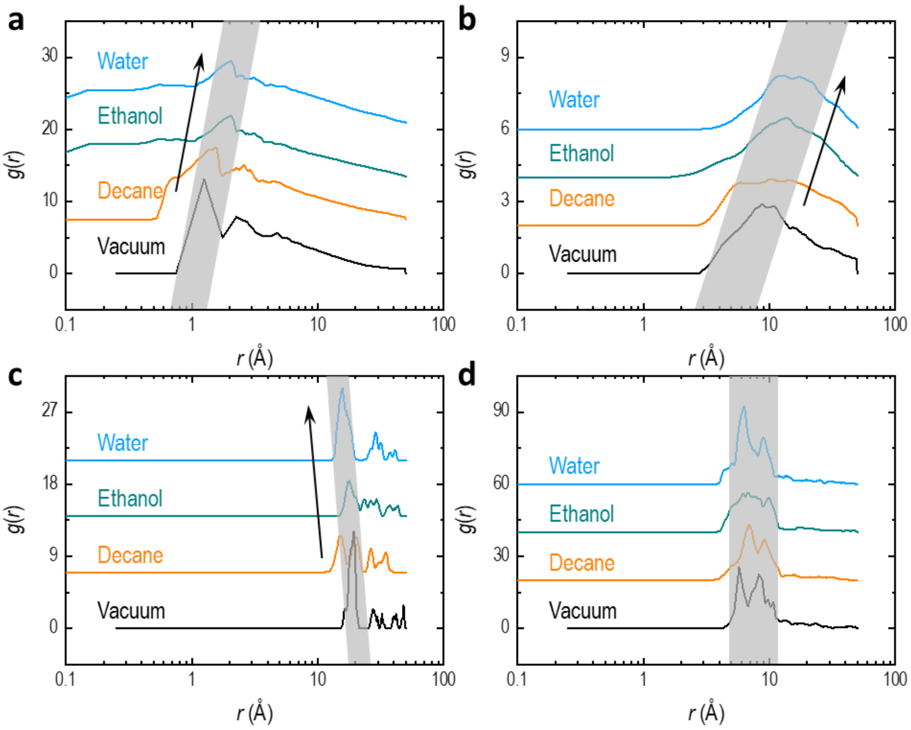

Figure S24. RDFs of inter-species when 200 solvent molecules are added. The simulation time is 2 ns. (a–d) RDFs of binder–binder (a), POSS–binder (b), POSS–POSS (c), and (d) fluorine–POSS, respectively. The arrows and shaded areas are to indicate the shifts of the radial distances of the first nearest inter-species neighbors.

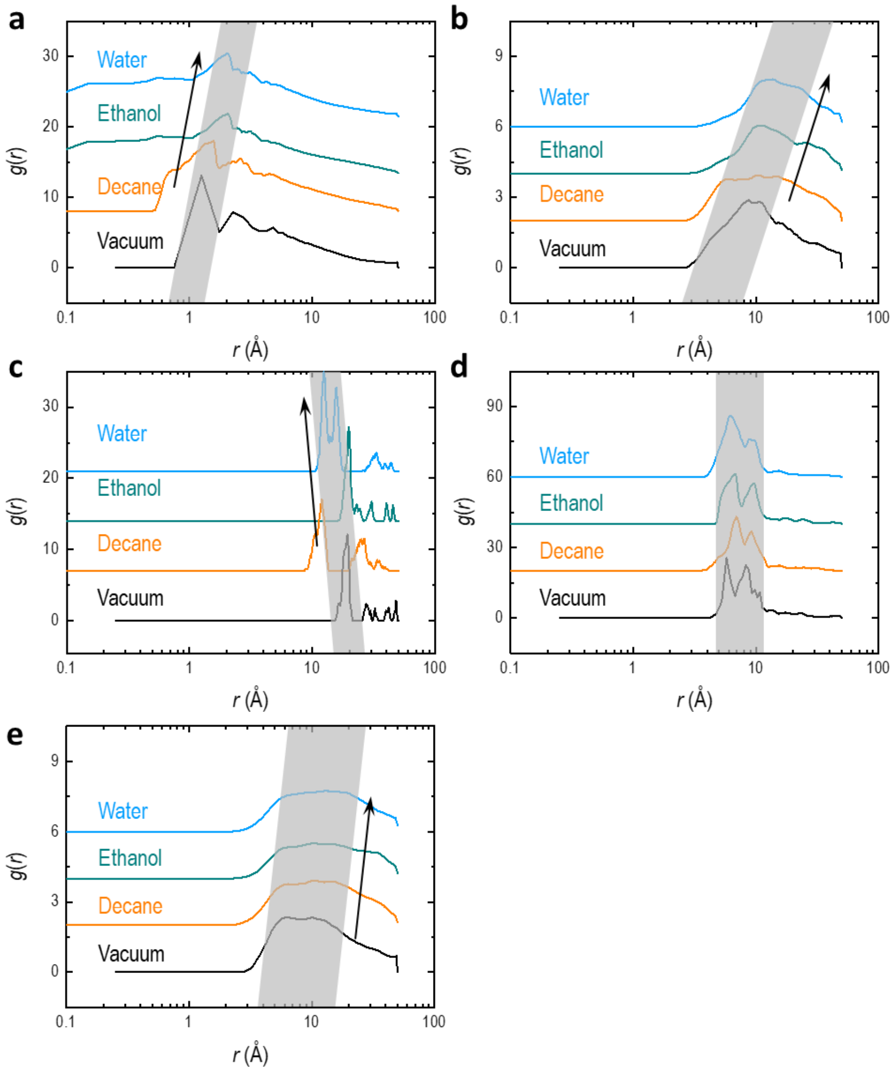

Figure S25. RDFs of inter-species when 6400 solvent atoms are added. The simulation time is 2 ns. The same number of atoms correspond to 200 *n*-decane, 711 ethanol, or 2133 water molecules, respectively. (a–e) RDFs of binder–binder (a), POSS–binder (b), POSS–POSS (c), fluorine–POSS (d), and (e) fluorine–binder, respectively. The arrows and shaded areas are to indicate the shifts of the radial distances of the first nearest inter-species neighbors.

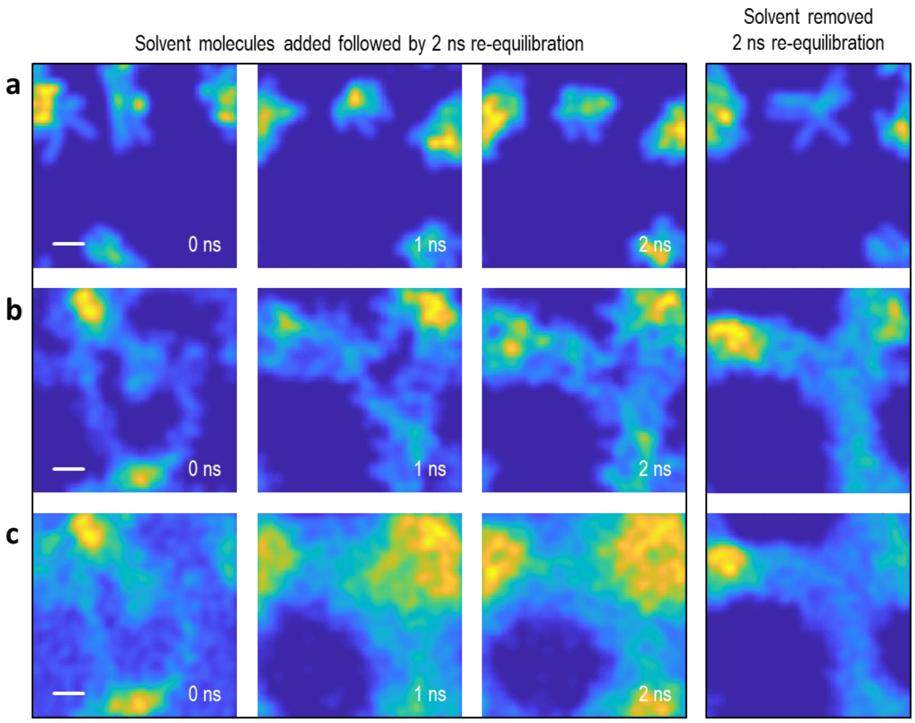

Figure S26. Projected mass distributions when water molecules are present. Blue-to-yellow (i.e., vacuum-to-mass aggregates) color coding is used, and the dark blue and light yellow correspond to vacuum and the aggregated mass, respectively: (a) POSS molecules, (b) binder molecules, and (c) POSS–binder composite (i.e., overlapped POSS, binder, and 2133 water molecules). The number of POSS and binder molecules are both 5. Scale bars are 10 Å.

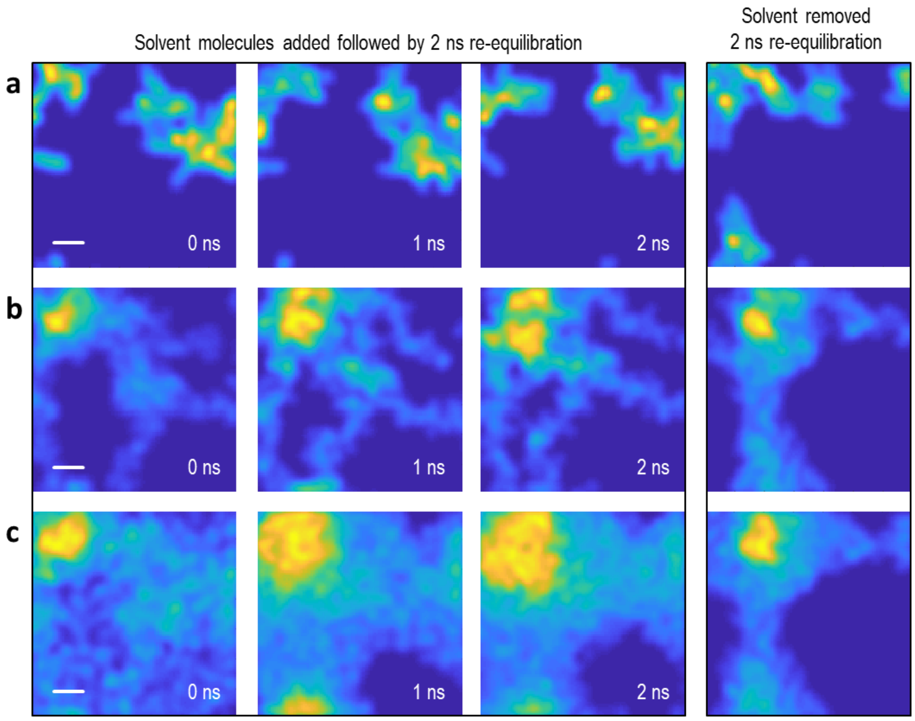

Figure S27. Projected mass distributions when ethanol molecules are present. Blue-to-yellow (i.e., vacuum-to-mass aggregates) color coding is used, and the dark blue and light yellow correspond to vacuum and the aggregated mass, respectively: (a) POSS molecules, (b) binder molecules, and (c) POSS–binder composite (i.e., overlapped POSS, binder, and 711 ethanol molecules). The number of POSS and binder molecules are both 5. Scale bars are 10 Å.

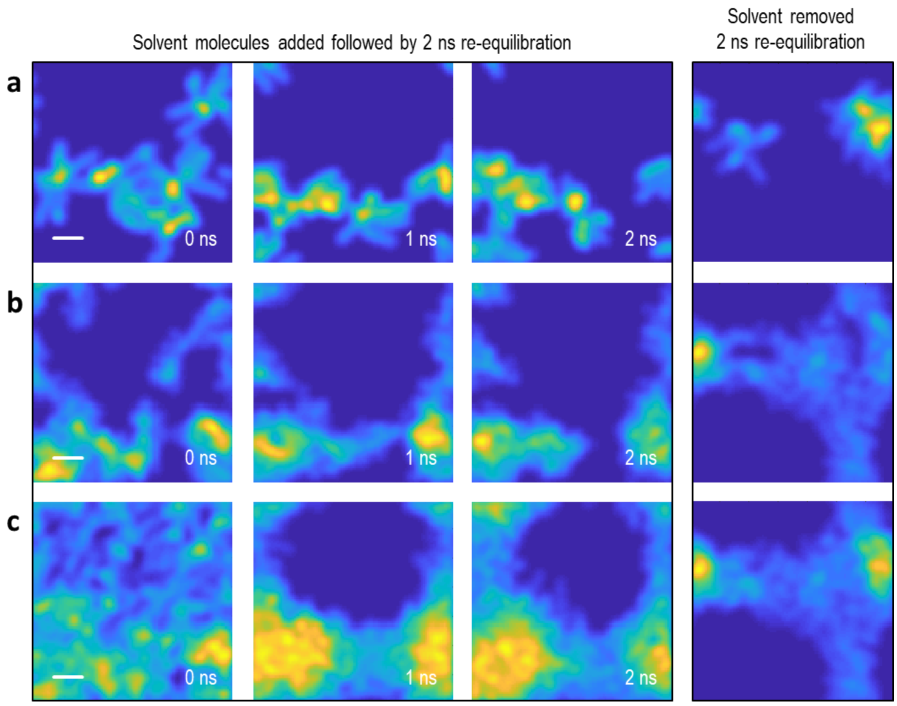

Figure S28. Projected mass distributions when decane molecules are present. Blue-to-yellow (i.e., vacuum-to-mass aggregates) color coding is used, and the dark blue and light yellow correspond to vacuum and the aggregated mass, respectively: (a) POSS molecules, (b) binder molecules, and (c) POSS–binder composite (i.e., overlapped POSS, binder, and 200 *n*-decane molecules). The number of POSS and binder molecules are both 5. Scale bars are 10 Å.

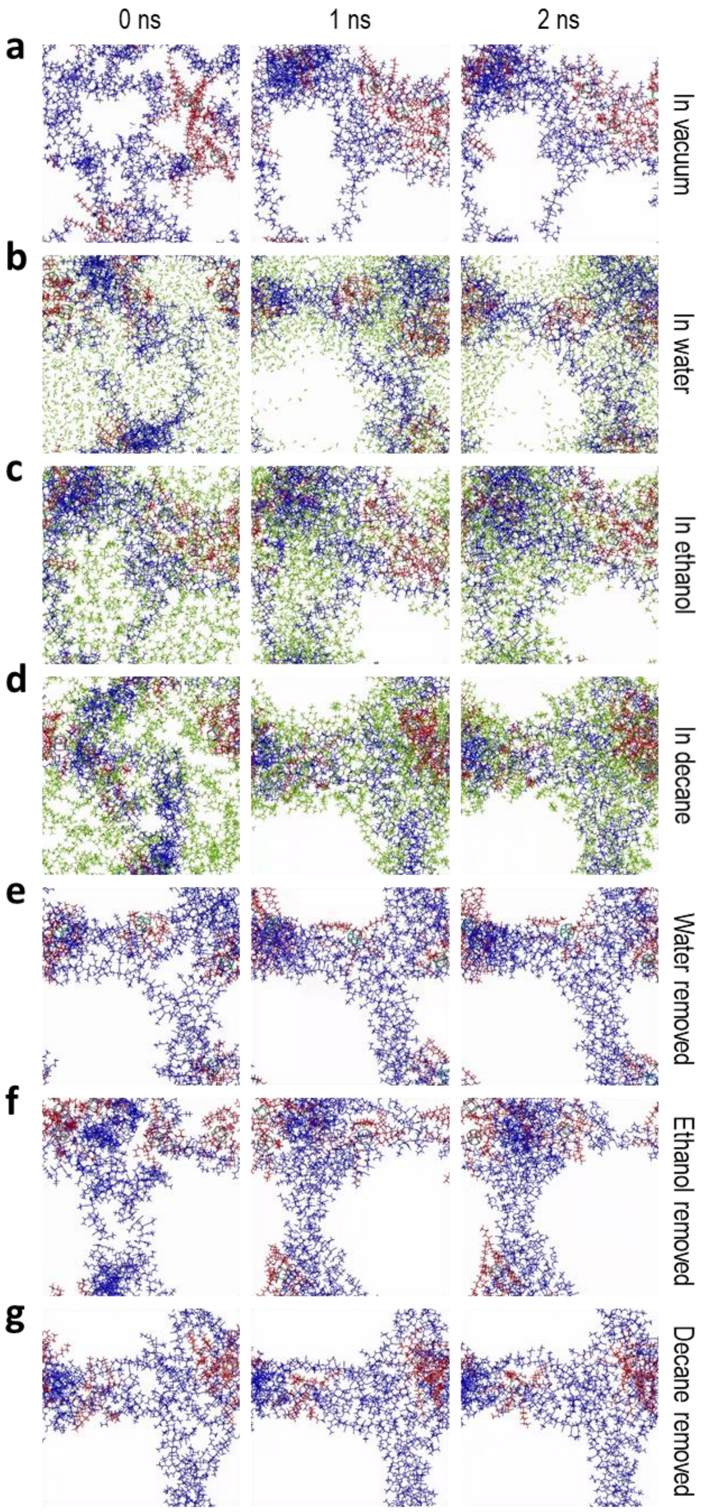

Figure S29. MD simulations with solvent molecules added and/or removed. The number of POSS and binder molecules are both 5. (a) Assembly in a vacuum. (b–d) Re-equilibrium when solvents are added: (b) 2133 water molecules; (c) 711 ethanol molecules; (d) 200 decane molecules. The total number of solvent atoms in each system is 6400. (e–g) Re-equilibrium in the vacuum when solvent molecules are completely removed. The dimension of the simulation cell is 70 × 70 × 70 Å^3. POSS and binder molecules are presented by red and blue, respectively.

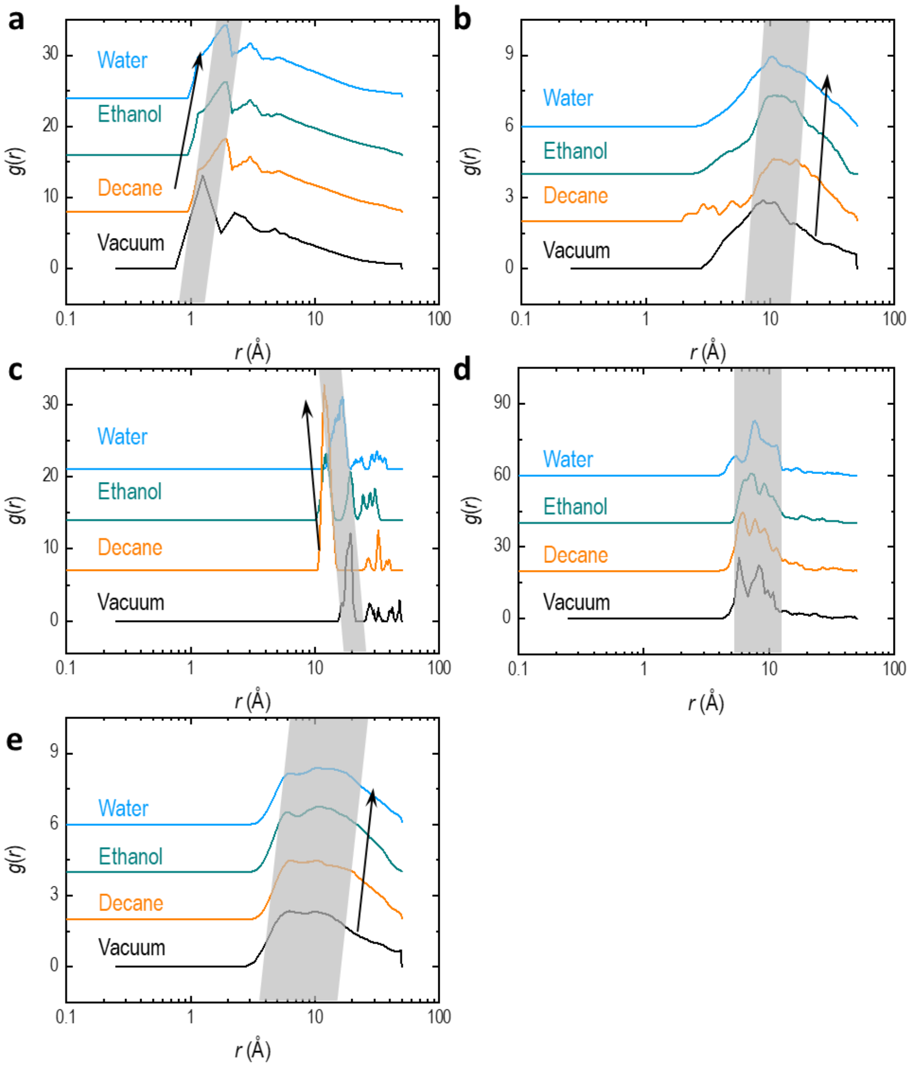

Figure S30. RDFs of inter-species when the solvent molecules are completely removed. The simulation time is 2 ns. (a–e) RDFs of binder–binder (a), POSS–binder (b), POSS–POSS (c), fluorine–POSS (d), and (e) fluorine–binder, respectively. The arrows and shaded areas are to indicate the shifts of the radial distances of the first nearest inter-species neighbors.

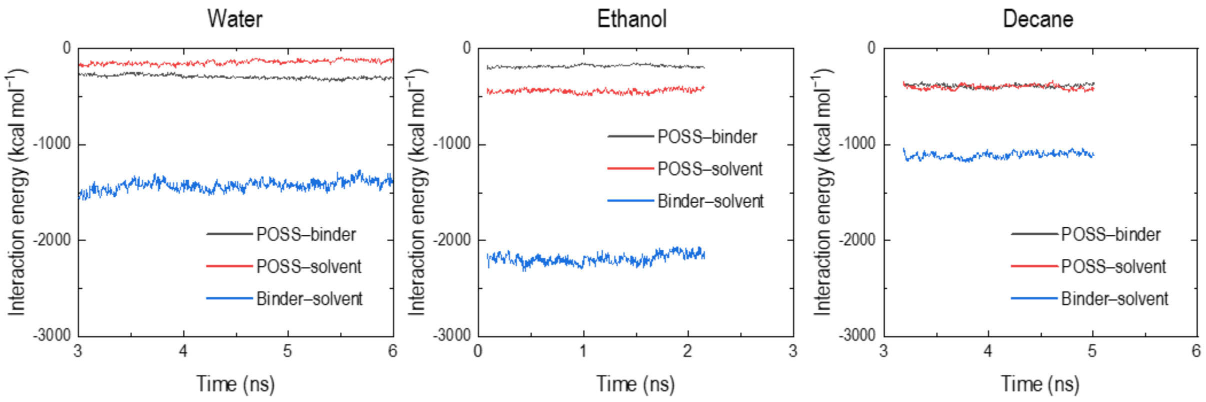

Figure S31. Interaction energy evolution assessed by MD simulations. The model system is 5×POSS + 5×pECA (polymeric binder). The POSS–binder and POSS–solvent interaction energies are comparable especially in decane. The solvent–binder interactions are more negative, indicating that the polymeric binder exerts a higher attractive force on the solvent than the POSS does. The interactions between the solvent and the binder have the following order: ethanol > water > decane with their interaction energies of about −2200, −1500, and −1100 in ethanol, water, and decane, respectively.

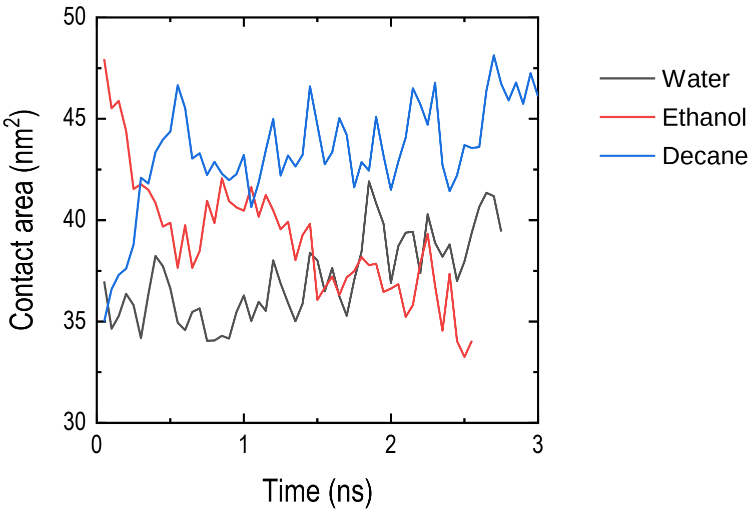

Figure S32. Contact area between the POSS and the biner components. The model system 5×POSS + 5×pECA was assessed by MD simulations. When the system was immersed in corresponding liquid, the contact area between the POSS and the binder components started to evolve. The contact area was larger when decane was the solvent, while a smaller contact area was observed in polar solvents (e.g., water, ethanol). This observation is consistent with the interaction energy between the POSS and the binder in solutions, e.g., −184.5 ± 12.5, −293.6 ± 20.9, and −390.5 ± 16.4 kcal mol^{-1} in ethanol, water, and *n*-decane, respectively.

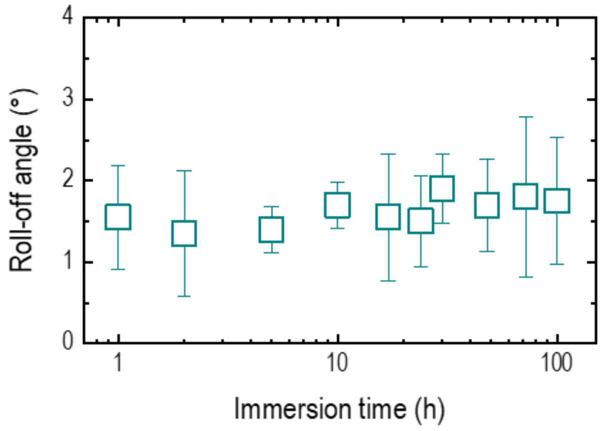

Figure S33. Roll-off angles of water. The super-repellent surface was immersed in ethanol for up to 100 h before being subjected to roll-angle measurement. The errors are standard deviations of >3 measurements.

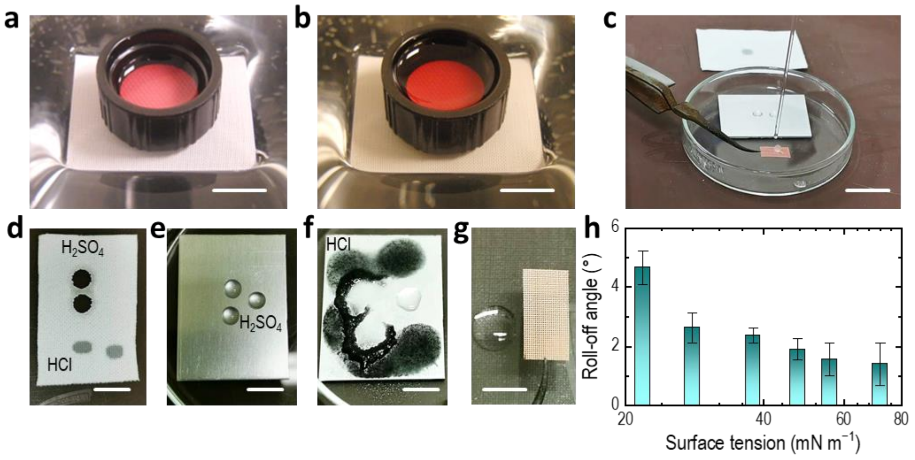

Figure S34. Chemical shielding test. (a, b) Super-repellent rafts floating with a load on 32 wt.% HCl (a) and (b) ~19 M NaOH. Scale bars are 7 mm. (c) Droplets of 98 wt.% H_2SO_4 impact on a super-repellent coating and roll off. Scale bar is 2 cm. (d) A piece of polyester fabric treated by two drops of 98 wt.% H_2SO_4 and 32 wt.% HCl, respectively. The photo was taken in ~3 min after the corrosive acids were dripped on the fabric. (e, f) An aluminum plate was treated by 3 drops of 98 wt.% H_2SO_4 (e) followed by 4 drops of 32 wt.% HCl (f). (g) The super-repellent surface after treatment shown in c. Scale bars are 1 cm in d–g. (h) Roll-off angles of water–ethanol mixtures after treatment shown in c. The errors are standard deviations of >3 measurements for each condition.

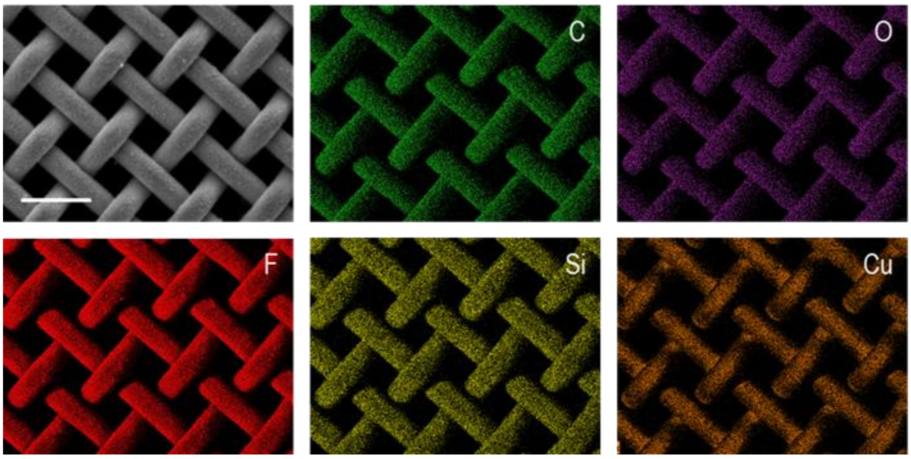

Figure S35. SEM image and EDX mapping of the super-repellent coating after 10 min immersion in 98 wt.% H_2SO_4. The elements imaged include C, N, O, F, and Si as displayed on the images. Scale bar is 200 μm and applies to all of the images.

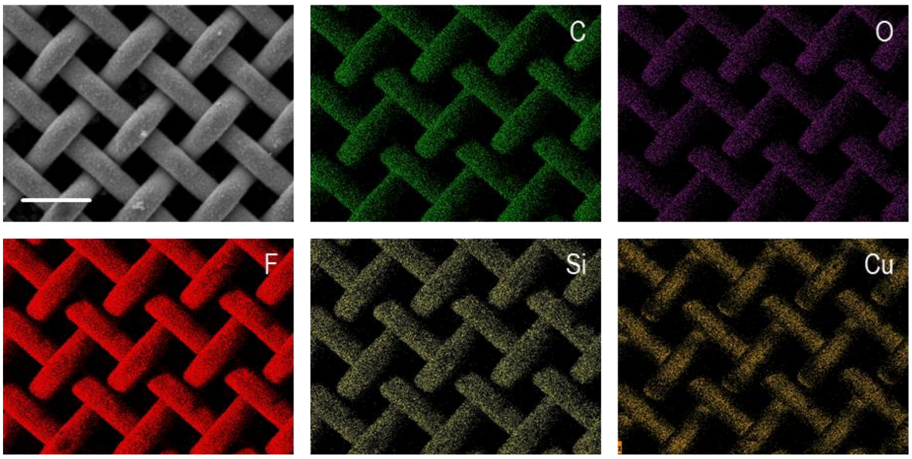

Figure S36. SEM image and EDX mapping of the super-repellent coating after 5 min heat treatment at 150°C. The elements imaged include C, N, O, F, and Si as displayed on the images. Scale bar is 200 μm and applies to all of the images.

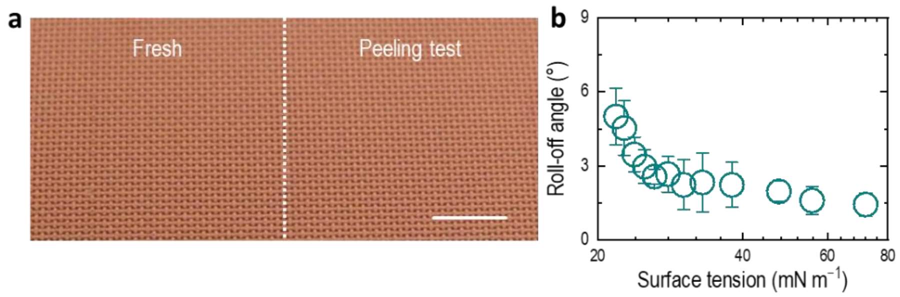

Figure S37. Peeling test. (a) A super-repellent surface before and after the peeling test. (b) Water–ethanol roll-off angles after peeling test. The errors are standard deviations of >3 measurements.

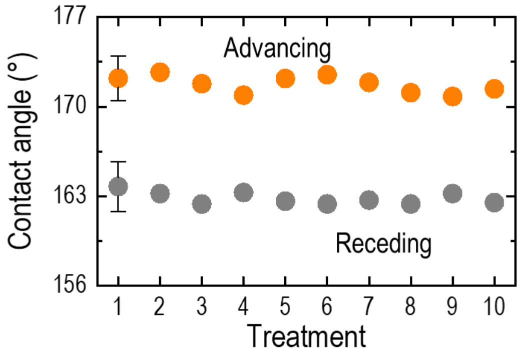

Figure S38. Water contact angles of the pre-treated super-repellent coatings. The type of treatment are labelled as: 1 = fresh coating without treatment (errors are standard deviations of >3 measurements); 2 = 5 min heat treatment at 150°C; 3 = 20 min UV–O_3 treatment; 4 = 60 s air plasma treatment; 5 = tape peeling (applied force ~10 N) test; 6 = 10 min immersion in ethanol; 7 = 10 min immersion in *n*-decane; 8 = 10 min immersion in dimethylformamide; 9 = 10 min immersion in 98 wt.% H_2SO_4; 10 = 10 min immersion in ~19 M NaOH.

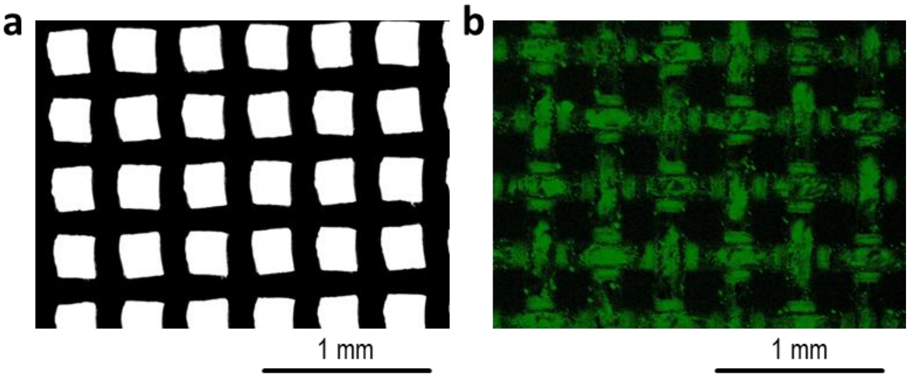

Figure S39. Fluorescence test. Florescent microscope image of (**a**) the super-repellent stainless steel mesh (70 × 70) and (**b**) the noncoated control after immersion in 1 mg mL^{-1} FITC in ethanol for 10 min.

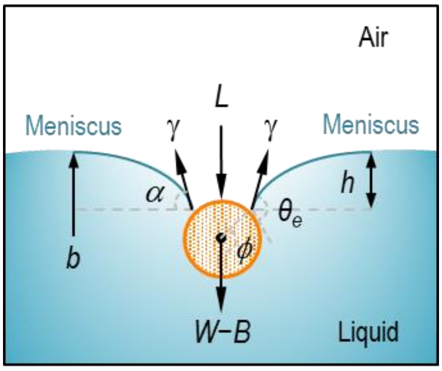

Figure S40. Forces acting on a metal wire (cross-section). Note the super-repellent mesh surface consisted of these metal wires. The schematic was adapted from the literature.[S14] L is the weight of the load, $W - B$ is the difference between the total weight and buoyancy. γ is the surface tension. θ_e is the local contact angle (i.e., equilibrium contact angle). ϕ is the angle between the gravitational direction and the triple-phase contact line. h is the height of the meniscus above the triple-phase contact line. b is the radius of the meniscus curvature. α is the angle between the horizontal plane and the surface tension. The order of the magnitude of surface tension acting at the metal wire surface is ~10^{-1} N m^{-1}, much greater than the other forces, i.e., weight (~10^{-7} N m^{-1}), buoyancy (~10^{-7} N m^{-1}), and the force acting at the triple-phase contact line due to higher liquid pressure than the atmosphere (~10^{-4} N m^{-1}). Therefore, the surface tension term determines the loading capacity.

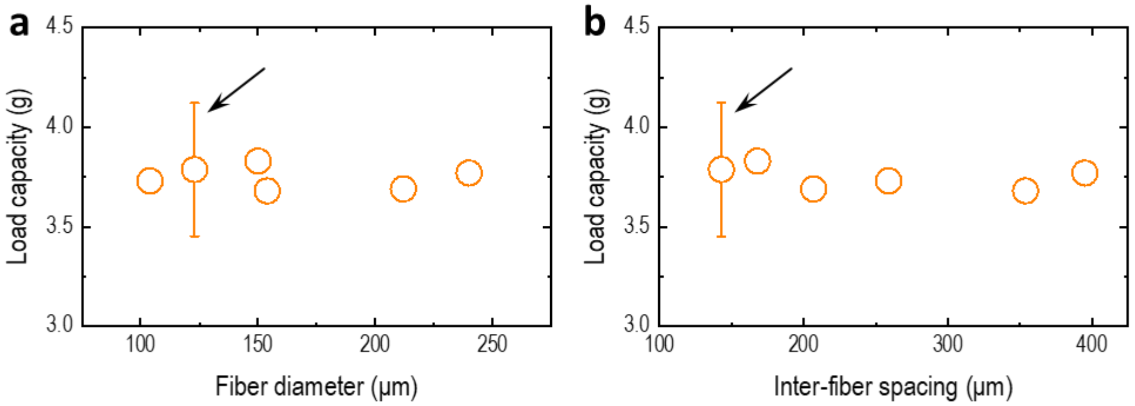

Figure S41. Effect of the substrate parameters on loading capacity on water. (**a**) Fiber diameter effect. (**b**) Inter-fiber spacing effect. The substrates are copper meshes (60 × 60, 80 × 80, and 100 × 100) and stainless steel meshes (40 × 40, 50 × 50, and 70 × 70). The datasets indicated by the arrow are that of the super-repellent raft prepared from 100 × 100 copper mesh. The diameter of the rafts are all ~2.54 cm in diameter.

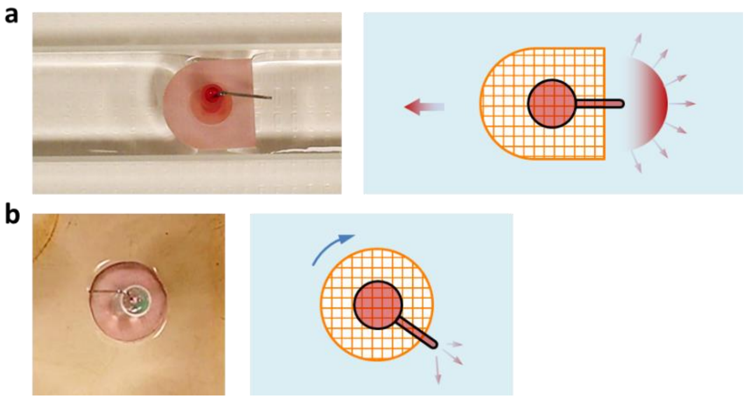

Figure S42. Locomotion control. (a) Linear motion confined by a narrow waterway. (b) Rotational motion on an open waterway. The diameters of the raft/boat are ~2.54 cm.

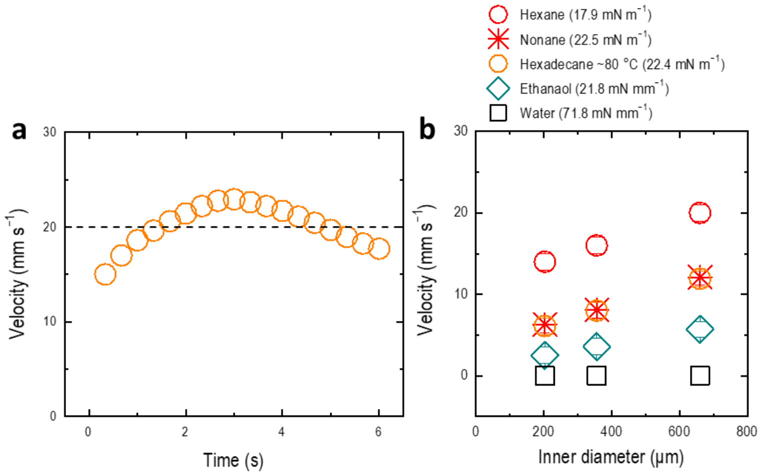

Figure S43. Velocity control of continuous linear motion. (a) Velocity as a function of time. The dashed line indicates the average velocity. (b) Controlling the average velocity by controlling the surface tension gradient, i.e., more fuel liquid released through capillary with a wider inner diameter, using lower surface tension liquids. The errors are standard deviations of >3 measurements.

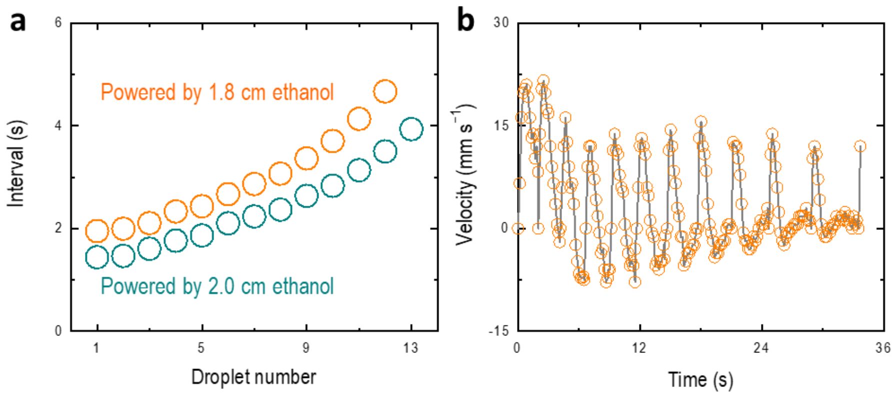

Figure S44. The velocity of pulsive locomotion. (a) The interval between each droplet as a function of the initial ethanol liquid loaded. (b) The velocity of typical pulsive locomotion, where the increased interval is a result of the decreased height of the loaded ethanol (i.e., fuel liquid).

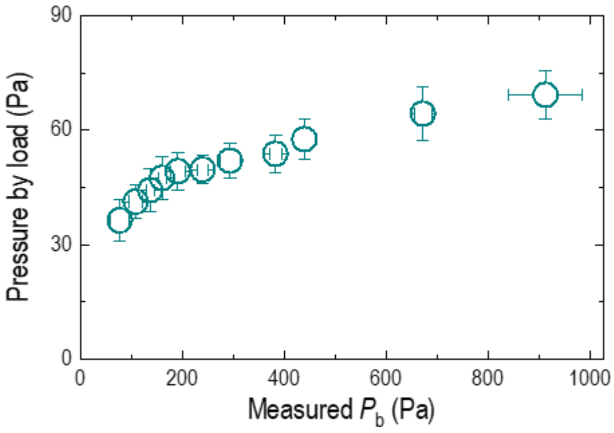

Figure S45. Pressure caused by the load. The X-axis represents the measured breakthrough pressure of water–ethanol mixtures on the super-repellent raft. The pressures applied by the maximum load were much smaller than the predicted, indicating no liquid break-in occurred at the maximum load. Errors are standard deviations of >3 measurements.

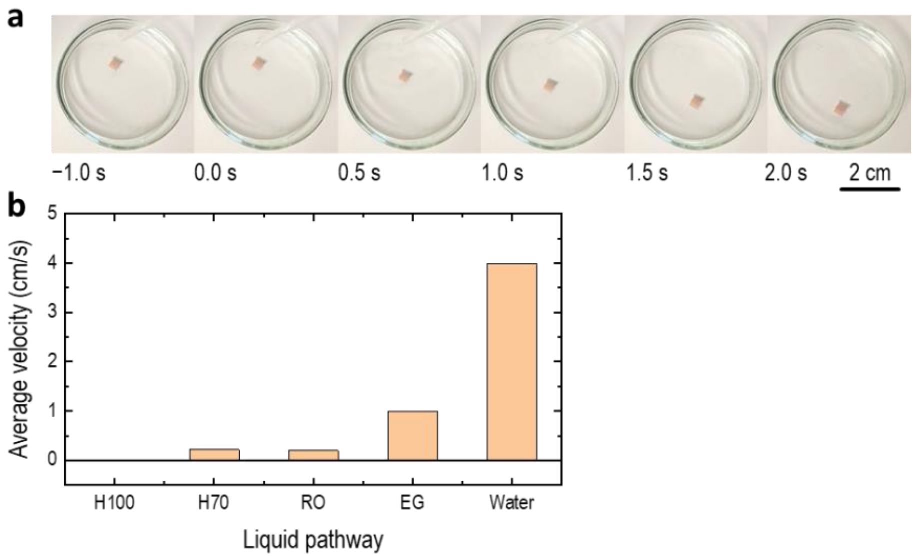

Figure S46. Super-repellent rafts travelling on different liquids powered by 10 μL ethanol. (**a**) Motion snapshots on the surface of ethylene glycol (EG). (**b**) Comparisons of average moving velocity on different liquids (with varied surface tension and viscosity). The dimensions of the super-repellent raft are 5 mm × 5 mm. H100 = honey; H70 = honey + 30 wt.% water; RO = rapeseed oil. Note that the viscosity values (at 20°C) are ~10^4, 70, 16, and 1 mPa·s for honey, RO, EG, and water, respectively, while their surface tensions (at 20°C) are 62, 34, 48, and 72 mN m^{-1} for honey, RO, EG, and water, respectively. For reference, the viscosity and surface tension of ethanol are ~1 mPa·s and 22 mN m^{-1} respectively. The results indicate that the diving force provided by the surface tension gradient could be dissipated by the viscosity of the liquid pathway.

Captions of Movie S1–S8

Movie S1. MD simulation of POSS–binder (5:10) at 298 K in a vacuum. The polymeric binders are presented in blue. The fluorinated alkyl chains are coded by red. The cage structures shown are the silicon–oxygen backbones of POSS. The video corresponds to a simulation time of 2 ns.

Movie S2. Bouncing ethanol droplet. A volume of ~5 μL of ethanol was dropped gently onto the surface from a height of about 7 mm above the surface and the process was recorded at 3900 fps. The ethanol droplet bounces off the surface and four bounces were observed. No break-in (liquid penetration) was observed during impact, indicating the robust super-repellency of the coating to this low surface tension liquid. The video plays at a speed of 30 fps.

Movie S3. MD simulation of POSS–binder (5:5) at 298 K in a vacuum. The polymeric binders are presented in blue. The fluorinated alkyl chains are coded by red. The cage structures shown are the silicon–oxygen backbones of POSS. The video corresponds to a simulation time of 2 ns.

Movie S4. Re-equilibrium of POSS–binder (5:5) at 298 K with ethanol molecules added. The polymeric binders are presented in blue. The fluorinated alkyl chains are coded by red. The cage structures shown are the silicon–oxygen backbones of POSS. The ethanol molecules (711, i.e., 6400 atoms) are coded in green. The video corresponds to a simulation time of 2 ns.

Movie S5. Re-equilibrium of POSS–binder (5:5) at 298 K with ethanol molecules removed. The polymeric binders are presented in blue. The fluorinated alkyl chains are coded by red. The cage structures shown are the silicon–oxygen backbones of POSS. The video corresponds to a simulation time of 2 ns.

Movie S6. Pulsive locomotion. A super-repellent raft (~2.54 cm in diameter) loaded with a container filled with ethanol. One end of the needle was immersed in the ethanol container while another was slightly suspended above the water surface. Once the low surface tension ethanol is released, the surface tension of water at the rear of the raft decreases, which drives the raft moving ahead. The video is real-time.

Movie S7. Continuous linear locomotion. A super-repellent raft (~2.54 cm in diameter) loaded with a container filled by ethanol. One end of the needle was immersed in the ethanol container while another was immersed in the water. Once the low surface tension ethanol is released, the surface tension gradient formed drives the raft moving ahead continuously. The video is real-time.

Movie S8. Rotational locomotion. A super-repellent boat (~2.54 cm in diameter) loaded with a container filled with ethanol. One end of the needle was immersed in the ethanol container while another was immersed in the water. Once the low surface tension ethanol is released, the surface tension gradient formed drives the raft moving rotationally. The video is real-time.

Supporting Reference S1–S14

[S1] Tuteja, A.; Choi, W.; Ma, M.; Mabry, J. M.; Mazzella, S. A.; Rutledge, G. C.; McKinley, G. H.; Cohen, R. E. Designing superoleophobic surfaces. *Science* **2007**, *318*, 1618–1622.

[S2] Mabry, J. M.; Vij, A.; Iacono, S. T.; Viers, B. D. Fluorinated polyhedral oligomeric silsesquioxanes (F-POSS). *Angew. Chem. Int. Ed.* **2008,** *47*, 4137–4140.

[S3] Scienomics, MAPS®, Paris, **2015**.

[S4] Lafayette, L.; Sauter, G.; Vu, L.; Meade, B. Spartan performance and flexibility: An HPC-cloud chimera, OpenStack Summit, Barcelona, October 27, **2016**. DOI: 10.4225/49/58ead90dceaaa.

[S5] Plimpton, S. Fast parallel algorithms for short-range molecular dynamics. *J. Comput. Phys.* **1995**, *117*, 1–19.

[S6] Swope, W. C.; Andersen, H. C.; Berens, P. H.; Wilson, K. R. A computer simulation method for the calculation of equilibrium constants for the formation of physical clusters of molecules: Application to small water clusters. *J. Chem. Phys.* **1982**, *76*, 637–649.

[S7] Sun, H.; Mumby, S. J.; Maple, J. R.; Hagler, A. T. An ab initio CFF93 all-atom force field for polycarbonates. *J. Am. Chem. Soc.* **1994**, *116*, 2978–2987.

[S8] Hockney, R. W.; Eastwood, J. W. Computer simulation using particles, Adam Hilger, NY, **1989**.

[S9] Willems, T. F.; Rycroft, C. H.; Kazi, M.; Meza, J. C.; Haranczyk, M. Algorithms and tools for high-throughput geometry-based analysis of crystalline porous materials. *Microp. Mesop. Mat.* **2012**, *149*, 134-141.

[S10] Humphrey, W.; Dalke, A.; Schulten, K. *J. Mol. Graphics* **1996**, *14*, 33–38.

[S11] Owens, D. K.; Wendt, R. C. Estimation of the surface free energy of polymers. *J. Appl. Polym. Sci.* **1969**, *13*, 1741–1747.

[S12] Tadmor, R. Line energy and the relation between advancing, receding, and Young contact angles. *Langmuir* **2004**, *20*, 7659–7664.

[S13] Pan, S.; Guo, R.; Xu, W. Investigating and biomimicking the surface wetting behaviors of ginkgo leaf. *Soft Matter* **2014**, *10*, 8800–8803.

[S14] Marmur, A.; Ras, R. H. A. The porous nano-fibers raft: Analysis of load-carrying mechanism and capacity. *Soft Matter* **2011**, *7*, 7382–7385.

Author Contributions

R.G. conceived the ideas, with the help of W.X. and S.P., designed, and led the project. All authors performed research and/or analyzed data with intellectual contributions. R.G. and S.P. drafted the manuscript with intellectual input from all authors. The authors declare no conflict of interest.
